# Fat mass and obesity-associated protein (FTO) mediated m^6^A modification of circFAM192A promoted gastric cancer proliferation by suppressing SLC7A5 decay

**DOI:** 10.1186/s43556-024-00172-4

**Published:** 2024-04-01

**Authors:** Xi Wu, Yuan Fang, Yunru Gu, Haoyang Shen, Yangyue Xu, Tingting Xu, Run Shi, Duo Xu, Jingxin Zhang, Kai Leng, Yongqian Shu, Pei Ma

**Affiliations:** 1 Department of Oncology, the First Affiliated Hospital of Nanjing Medical University, Nanjing, 210029 People’s Republic of China; 2 Department of General Surgery, Affiliated People’s Hospital of Jiangsu University, Zhenjiang Clinic School of Nanjing Medical University, Zhenjiang, People’s Republic of China; 3 Department of Medical Informatics, the First Affiliated Hospital of Nanjing Medical University, Nanjing, 210029 People’s Republic of China; 4 Jiangsu Key Lab of Cancer Biomarkers, Prevention and Treatment, Nanjing Medical University, Nanjing, China

**Keywords:** Gastric Cancer (GC), Fat mass and obesity-associated protein (FTO), N6-methyladinosine (m^6^A), Circular RNA (circRNA), Solute carrier family 7 member 5 (SLC7A5)

## Abstract

Gastric cancer (GC) is a common malignant tumor worldwide, especially in East Asia, with high incidence and mortality rate. Epigenetic modifications have been reported to participate in the progression of gastric cancer, among which m^6^A is the most abundant and important chemical modification in RNAs. Fat mass and obesity-associated protein (FTO) is the first identified RNA demethylase but little is known about its role in gastric cancer. In our study, data from TCGA and clinical samples showed that FTO was highly expressed in gastric cancer tissues. Kaplan–Meier plotter suggested that patients with the high level of FTO had a poor prognosis. In vitro and in vivo experiments confirmed the role of FTO in promoting gastric cancer cell proliferation. Mechanistically, we found that FTO bound to circFAM192A at the specific site and removed the m^6^A modification in circFAM192A, protecting it from degradation. CircFAM192A subsequently interacted with the leucine transporter solute carrier family 7 member 5 (SLC7A5) and enhancing its stability. As a result, an increased amount of SLC7A5 was on the membrane, which facilitated leucine uptake and activated the mTOR signaling pathway. Therefore, our study demonstrated that FTO promoted gastric cancer proliferation through the circFAM192A/SLC7A5 axis in the m^6^A-dependent manner. Our study shed new light on the role of FTO in gastric cancer progression.

**Supplementary Information:**

The online version contains supplementary material available at 10.1186/s43556-024-00172-4.

## Introduction

Gastric cancer (GC) poses a major threat to global health for its high incidence and mortality rate, and the disease burden is mainly in China where approximately 50% of newly diagnosed cancer cases and cancer deaths occur [1]. With the emergence of targeted agents, a considerable percentage of patients has been benefited. However, GC is highly heterogeneous and current targeted therapy options are still limited. Therefore, further investigation into the development of GC are crucial for GC prevention and treatment.

N6-methyladenosine (m^6^A) is the most prevalent chemical modification in RNAs and undergoes dynamic regulation [2]. The m^6^A modification is mainly written by the methyltransferase complex, of which methyltransferase 3 (METTL3) is the important catalytic component. Fat mass and obesity-associated protein (FTO) and AlkB Homolog 5 (ALKBH5) are common erasers that remove m^6^A from RNAs. Moreover, there is also a kind of protein that act as m^6^A reader, such as YTH N6-methyladenosine RNA binding protein- “YTHDCs”/ “YTHDFs” and the insulin-like growth factor 2 mRNA-binding protein- “IGF2BPs”. Plenty of studies have revealed that m^6^A is a double-edged sword in tumorigenesis, both acting as a cancer promoter or suppressor. For example, the m^6^A modification in YAP mRNA installed by METTL3 promoted YAP expression and caused the metastasis of non-small-cell lung carcinoma [3]; The decreased m^6^A level in LYPD1 mRNA caused its degradation and weakened the malignant behaviors of hepatocellular carcinoma [4]. As the first identified demethylase, FTO has been reported participating in tumorigenesis as well, such as melanoma, ovarian, renal cell carcinoma [5–7], but there are knowledge gaps about its role in GC.

Circular RNAs (circRNA) are generated through back-splicing from the linear transcripts and are characterized by their stability [8]. CircRNAs have been reported to function as transcriptional regulators, microRNA sponges [9, 10], protein templates, decoys, scaffolds, and recruiters [11, 12]. The m^6^A modification in circRNAs has the potential to facilitate the translation of circRNAs into proteins, accelerate their degradation rate, and promote their nuclear export [13, 14]. Several studies have investigated the relationship between m^6^A modification and circRNAs in cancer development [15–17], but our knowledge about m^6^A and circRNA in GC is limited and requires further exploration.

Amino acid transporters are located in membranes and transport amino acids. The solute carrier superfamily is the largest group, accounting for about 20% of all membrane transporters [18]. Solute carrier family 7 member 5 (SLC7A5) is one of the system L amino acid transporters that responsible for leucine uptake [19]. High cellular leucine concentration activates the mTOR pathway and promotes cell proliferation [20]. SLC7A5 is known to be highly expressed in many cancers [21, 22]. In KRAS-mutant colorectal cancer cells, SLC7A5 maintained intracellular amino acid level and activated mTOR pathway, which finally supported cell proliferation [23]; in small cell lung cancer, increased SLC7A5 promoted cell growth [24]. However, the current research on SLC7A5 in GC is insufficient.

In our study, we demonstrated the oncogenic role of FTO in GC and investigated the underlying mechanism. In detail, FTO bound to circFAM192A and removed the m^6^A modification in it, which enhanced circFAM192A stability. Upregulated circFAM192A inhibited SLC7A5 degradation and therefore more SLC7A5 located in the membrane, which caused leucine uptake and the mTOR pathway activation. Hence, FTO promoted GC proliferation through the circFAM192A/SLC7A5 axis and FTO could serve as a therapeutic target for GC patients.

## Results

### FTO was upregulated in GC and its high expression predicted a poor prognosis

**Fig. 1:**
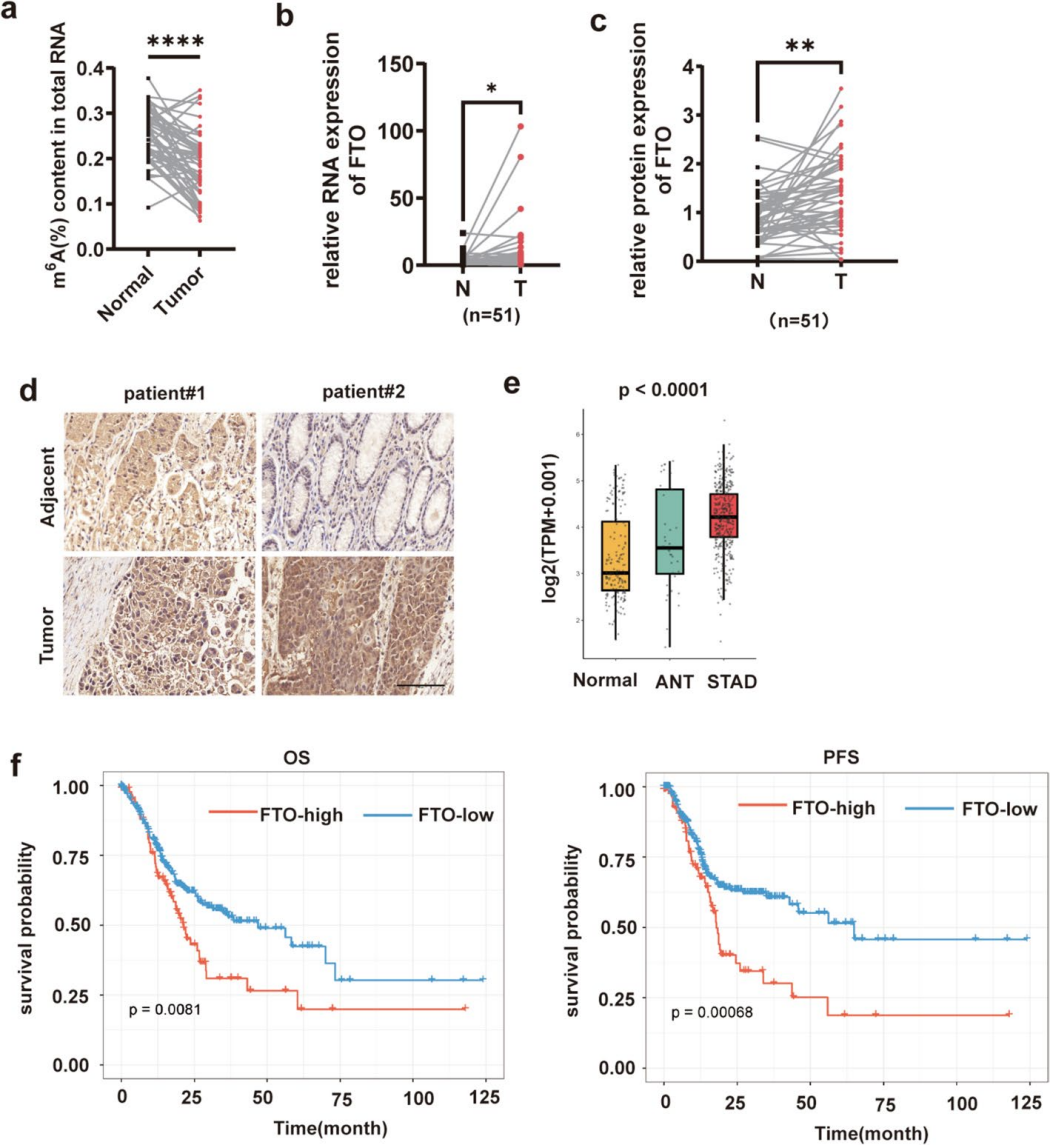
FTO was upregulated in GC and predicted a poor prognosis. **a** ELISA assays showed the m^6^A level in gastric cancer tissues and normal tissues; **b** QPCR assays displayed the FTO mRNA level in 51 pairs of GC samples from patients; **c** The chart exhibited the level of FTO protein in GC samples; **d** Representative IHC images of paired GC samples with FTO antibody, the scale bar is 20 μm; **e** The expression pattern of FTO in STAD, ANT and normal tissues with data from TCGA and GTEx database; **f** The OS (left) and PFS (right) curve of GC patients with different FTO expression drawn with data from TCGA. Quantitative data from three independent experiments are shown as the mean ± SD (error bars). *P < 0.05, **P < 0.01, ***P < 0.001 (Student’s t-test). STAD: stomach adenocarcinoma; ANT:adjacent normal tissues

To evaluate the m^6^A expression pattern in GC, we conducted colorimetric ELISA assays in 51 pairs of GC tissues and adjacent normal tissues. As shown in Fig. 1a, the m^6^A level was lower in GC tissues. Further, to figure out which m^6^A enzyme was associated with the decreased m^6^A level, qPCR assays were performed to assess the expression of METTL3, ALKBH5 and FTO. It was observed that only the level of FTO mRNA was significantly higher in GC samples than adjacent normal tissues (Fig. 1b, Fig. S1a). What’s more, we used the online tool GEPIA (http://gepia.cancer-pku.cn/) and found that the expression of METTL3 and ALKBH5 appeared no significant difference between GC and normal tissues (Fig. S1b). While the transcriptome data from the TCGA data portal [25] and GTEx database [26] showed that the level of FTO mRNA was significantly higher in stomach adenocarcinoma (STAD) samples than adjacent normal tissues (ANT) (Fig. 1e). Similarly, the protein level of FTO was increased in GC tissues (Fig. 1c,d, Fig. S1c).

To investigate the relationship between FTO expression and the survival time, we analyzed the survival data including overall survival (OS) and progression-free survival (PFS) from the TCGA data portal. Results showed that patients with high FTO expression had shorter OS and PFS (Fig. 1f). In summary, these results indicated that FTO was upregulated in GC tissues and its high expression was a negative signal for prognosis.

### FTO promoted GC proliferation in vitro and in vivo

**Fig. 2:**
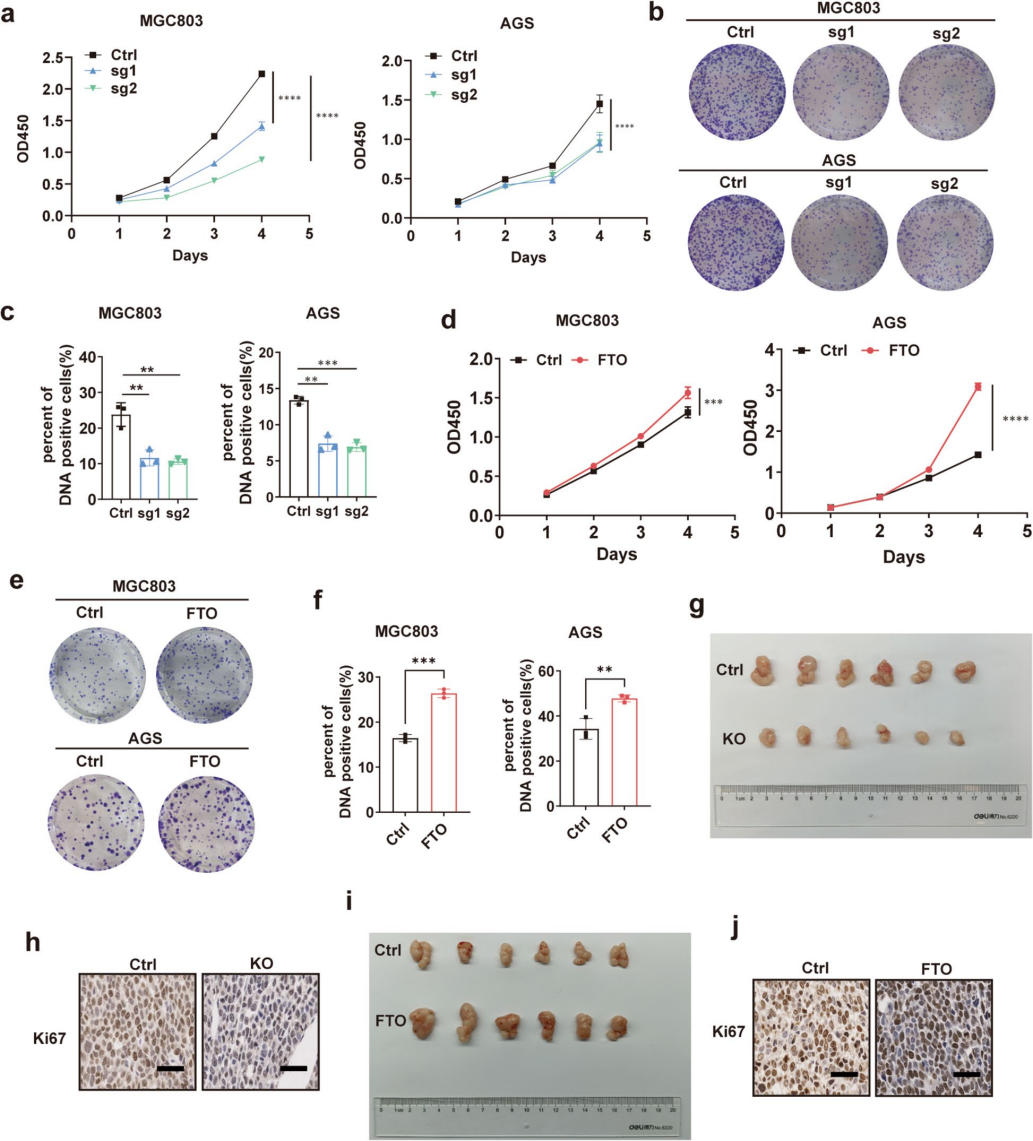
FTO promoted GC proliferation in vitro and in vivo. **a** CCK8 assays showed the growth rate of FTO knockout and controlled cells; **b** Colony formation assays showed clones grown from FTO knockout and controlled cells; **c** EdU assays exihibited the percent of DNA positive cells in FTO knockout and controlled cell lines; **d** The growth rate of FTO overexpressed and controlled cells evaluated by CCK8 assays; **e** Colony formation assays showed clones grown from FTO overexpressed and controlled cells; **f** The percent of DNA positive cells in FTO overexpressed or controlled cell lines in EdU experiments; **g** Tumors from mice injected with FTO knockout MGC803 cells and controlled cells; **h** The expression of Ki67 in tumors from FTO knockout group in IHC images, the scale bar is 20 μm; **i** Tumors from mice injected with FTO overexpressed MGC803 cells and controlled cells; **j** IHC images showed the expression of Ki67 in tumors from FTO overexpressed group, the scale bar is 20 μm. Quantitative data from three independent experiments are shown as the mean ± SD (error bars). *P < 0.05, **P < 0.01, ***P < 0.001 (Student’s t-test)

To explore the role of FTO in GC progression, we first knocked out FTO in MGC803 and AGS cell lines (Fig. S2a). CCK8 assays showed that knocking out FTO significantly inhibited the growth rate of GC cells (Fig. 2a). Colony formation assays showed that cells with FTO knockout grew into fewer and smaller clones (Fig. 2b, Fig. S2d). Similarly, it was observed in EdU assays that the percent of DNA-positive cells was decreased when FTO was knocked out (Fig. 2c, Fig. S2f). On the contrary, overexpressing FTO enabled GC cells to grow faster (Fig. 2d-f, Fig. S2b,c,e). However, transwell assays and wound healing assays showed that FTO had no effect on cell migration (Fig. S3).

To explore the in vivo effect of FTO on GC proliferation, we injected MGC803 cells with FTO knockout or overexpressed and untreated cells into nude mice subcutaneously. It was shown that the growth rate of tumors in the FTO knockout group was slower and tumors were smaller and lighter (Fig. 2g, Fig. S2g,h), while opposite results were detected in the FTO overexpressed group (Fig. 2i, Fig. S2i,j). IHC assays indicated that Ki67, the biomarker of proliferation was downregulated in tumors from the FTO knockout group (Fig. 2h), but increased in FTO overexpressed tumors (Fig. 2j).

Overall, these results demonstrated that FTO promoted GC proliferation both in vitro and in vivo.

### CircFAM192A was the downstream molecule of FTO

**Fig. 3:**
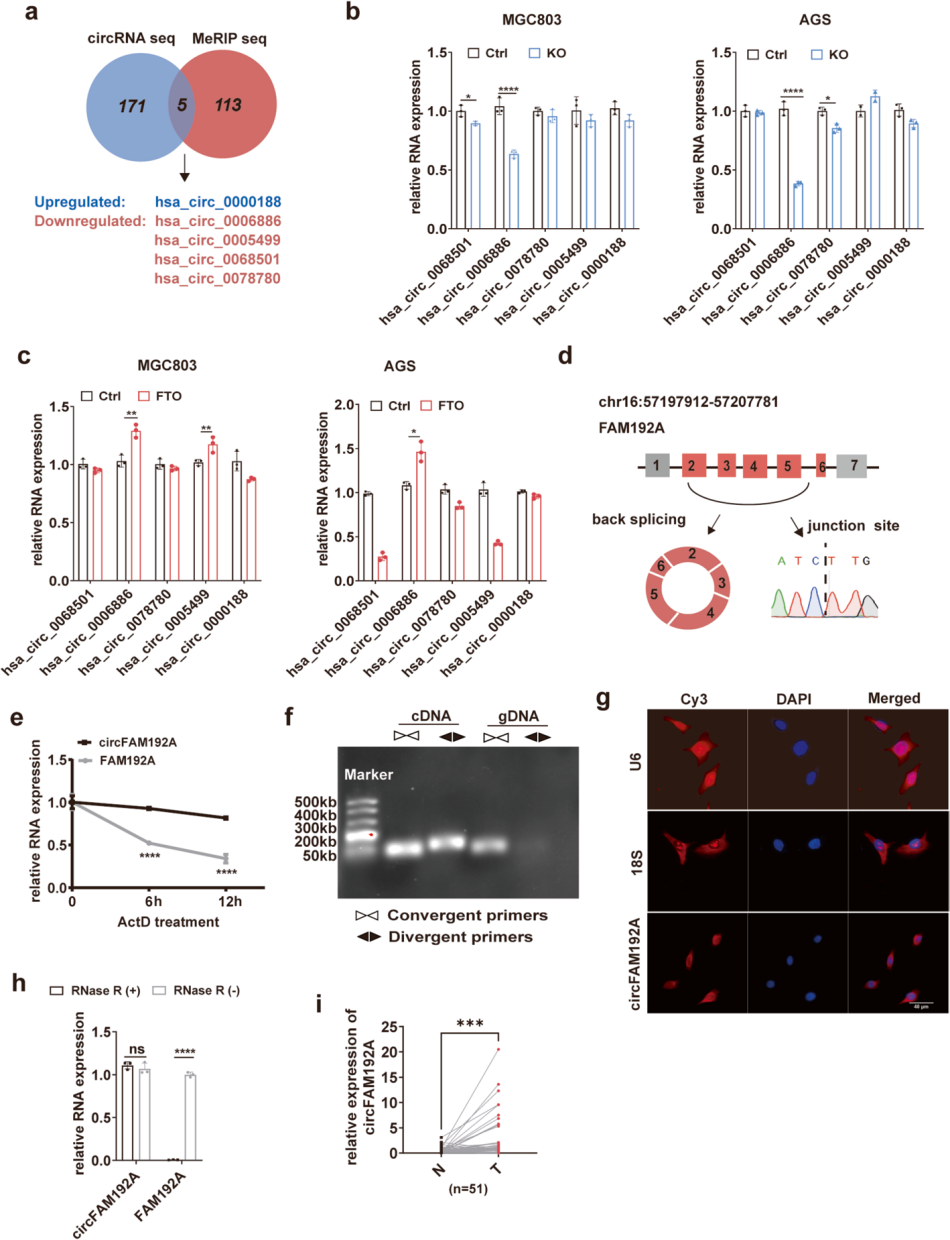
The identification and characteristic of circFAM192A. **a** The Venn diagram depicted overlapping circRNAs between MeRIP seq and circRNA seq; **b** The alteration of candidate cirRNAs upon FTO knockout; **c** The alteration of candidate cirRNAs upon FTO overexpression; **d** The diagrammatic structure of circFAM192A and Sanger sequencing validation for the junction site of circFAM192A; **e** The level of circFAM192A and linear FAM192A after Actinomycin D treatment (10 μg/ml) for 0, 6, 12 h; **f** Northern blotting showed PCR products amplified from cDNA/gDNA template using divergent primers and convergent primers; **g** The location of circFAM192A determined by FISH assays (scale bar, 40 μm). **h** The level of circFAM192A and linear FAM192A after RNase R treatment (5U for 1 g RNA); **i** QPCR assays displayed the expression of circFAM192A in gastric cancer tissues and normal tissues. Quantitative data from three independent experiments are shown as the mean ± SD (error bars). *P < 0.05, **P < 0.01, ***P < 0.001 (Student’s t-test)

As previous studies have reported the relationship between m^6^A modifications and circRNAs in cancerogenesis [13, 14], we hypothesized that FTO may regulate circRNA expression through the m^6^A dependent manner. CircRNA sequencing and m^6^A-modified RNA immunoprecipitation (MeRIP) sequencing were performed with FTO knockout MGC803 cells and untreated MGC803 cells (Fig. S4). CircRNA-seq indicated that 13 circRNAs were significantly upregulated (fold change > 2), while 163 circRNAs were significantly downregulated (fold change < 0.3) in FTO knockout MGC803 cells. It was detected in MeRIP-seq that 118 circRNAs were more methylated (fold change > 1.4) when FTO was knocked out. Through overlapping differentially expressed circRNAs and methylated circRNAs, we identified four downregulated cirRNAs, hsa_circ_0006886, hsa_circ_0005499, hsa_circ_0068501, hsa_circ_0078780, and one upregulated circRNA, hsa_circ_0000188 (Fig. 3a). We further found that only hsa_circ_0006886 was consistently downregulated or upregulated in both cell lines when FTO was knocked out or overexpressed (Fig. 3b,c). Therefore, we presumed that hsa_circ_0006886 was under FTO regulation.

Hsa_circ_0006886 was back spliced from exons 2–6 of protein coding gene, FAM192A (Fig. 3d), and thus named as circFAM192A. Sanger sequencing confirmed the junction site between exon 2 and exon 6 (Fig. 3d). Consistently, circFAM192A could be amplified from cDNA and gDNA by convergent primers, but only from cDNA by divergent primers, validating that circFAM192A truly had a circular structure (Fig. 3f). In addition, as shown in Fig. 3e and Fig. 3h, compared to linear transcripts, RNase R and ActD had little effect on circFAM192A expression, both implying that circFAM192A was stable. Moreover, FISH assays suggested that circFAM192A was mainly distributed in the cytoplasm (Fig. 3g). What’s more, we valuated the expression of circFAM192A in our cohort and found that circFAM192A was highly expressed in GC (Fig. 3i).

### FTO regulated circFAM192A expression through the m^6^A dependent manner

**Fig. 4:**
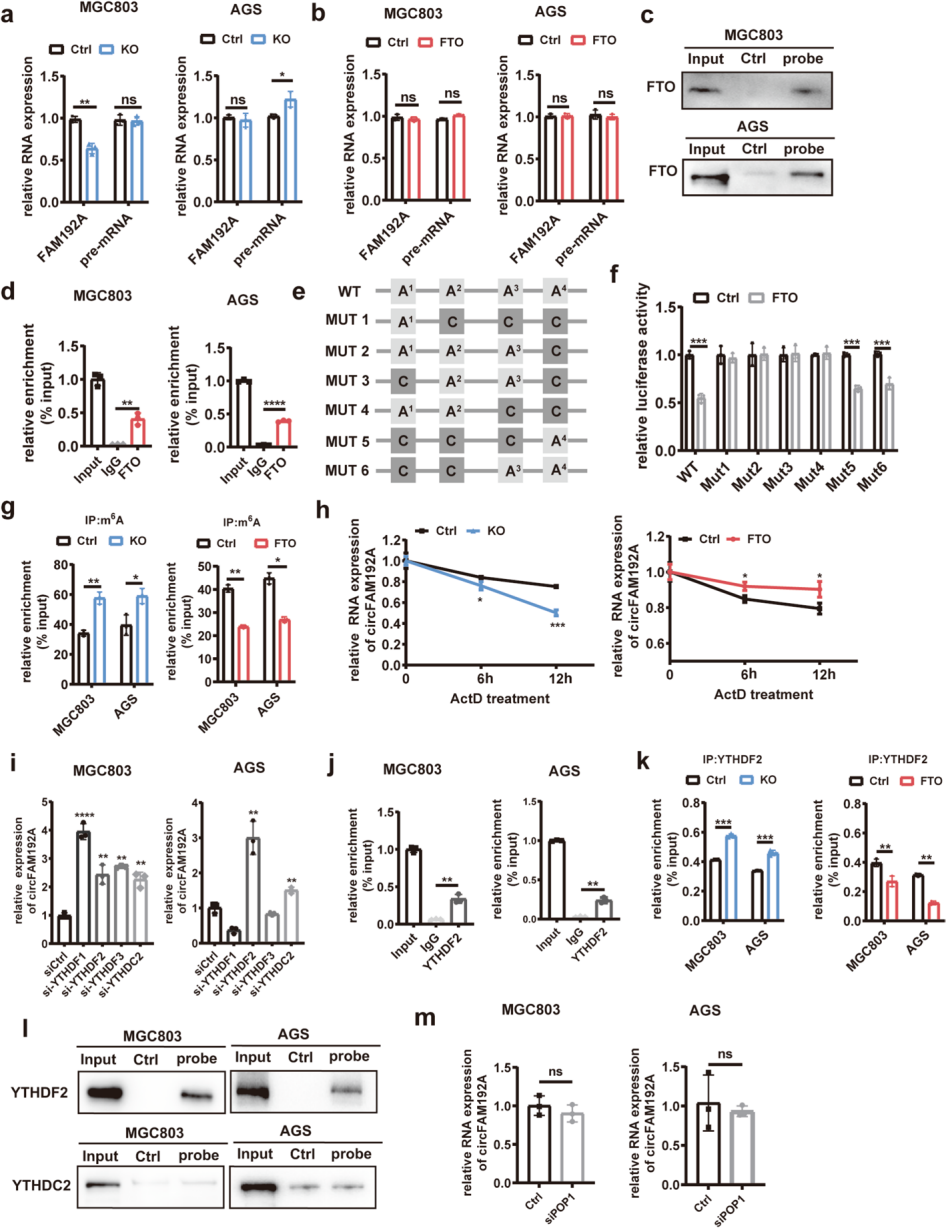
FTO regulated circFAM192A expression through the m^6^A dependent manner. **a** The expression of pre-mRNA and linear FAM192A upon FTO knockout; **b** The expression of pre-mRNA and linear FAM192A upon FTO overexpression; **c** Pull down assays exhibited the quantity of FTO protein captured by circFAM192A targeting probes and negative control probes; **d** CircFAM192A captured by anti-FTO antibody in RIP assays; **e** Drawing of luciferase plasmids with different mutant binding site; **f** The relative luciferase activity in HEK-293 T cells transfected with plasmids containing different mutant m^6^A sites; **g** The m.^6^A abundance in circFAM192A upon FTO knockout or overexpression; **h** The stability of circFAM192A under the condition of FTO knockout (left) and overexpression (right) in ActD treatment assays; **i** QPCR assays showed the level of circFAM192A when knocking down YTHDF1/2/3, YTHDC2; **j** The enrichment between circFAM192A and anti-YTHDF2 antibody in RIP assays; **k** RIP assays showed the level of circFAM192A captured by anti-YTHDF2 antibody after knocking out or ovexpressing FTO; **l** Pull down assays showed the level of YTHDF2 or YTHDC2 captured by circFAM192A targeting probes and negative probes; **m** QPCR assays showed the level of circFAM192A when knocking down POP1. Quantitative data from three independent experiments are shown as the mean ± SD (error bars). *P < 0.05, **P < 0.01, ***P < 0.001 (Student’s t-test)

To clarify the mechanism of FTO regulating circFAM192A expression, we first investigated whether circFAM192A influenced FTO expression. It was observed that knocking down or overexpressing circFAM192A had no effect on FTO expression (Fig. S5a). Then, we try to explore whether FTO influenced the production of circFAM192A. However, the pre-mRNA and linear FAM192A expression was inconsistent in MGC803 and AGS cells upon FTO knockout (Fig. 4a) and no significant alteration was observed upon FTO overexpression (Fig. 4b). Further, we performed pull down assays with a specific biotin labeled probe targeting the junction cite of circFAM192A. It was observed that more FTO protein was captured by the specific probe than the negative control one (Fig. 4c). Similarly, more circFAM192A was enriched with FTO antibody than IgG in RIP experiments (Fig. 4d). These results revealed that FTO bound to circFAM192A directly. In order to determine the binding site between FTO and circFAM192A, we searched SCRAMP software (http://www.cuilab.cn/sramp) and RMBase (https://rna.sysu.edu.cn/rmbase/index.php). There were four overlapping potential sites with high possibility (Fig.S5b). We then generated recombinational luciferase reporter plasmids containing mutated circFAM192A sequence (Fig. 4e, Fig. S5b). As shown in Fig. 4f, the ectopic expression of FTO weakened the luciferase activity in 293 T cells transfected with mut 5 and 6 plasmids, similar to that of WT group, which indicated that FTO bound to circFAM192A at site 4. Corresponding to the MeRIP seq results, MeRIP-qPCR confirmed that the m^6^A-specific antibody captured more circFAM192A in FTO knockout cells while captured less circFAM192A in FTO overexpressed cells (Fig. 4g), suggesting that FTO erased m^6^A modification in circFAM192A. It has been reported that the m^6^A modification in circRNAs may impact their stability. ActD treatment assays indicated that knocking out FTO accelerated the degradation of circFAM192A, but overexpressing FTO enhanced its stability (Fig. 4h), demonstrating that FTO played a protective role in circFAM192A decay.

Previous studies have suggested that m^6^A readers recognized m^6^A modifications in RNAs and regulated their expression. YTHDF1/2/3 and YTHDC2 were reported to relate with circRNA degradation [27], but only knocking down YTHDF2 and YTHDC2 caused the increased circFAM192A expression in both cell lines (Fig. 4i). Following pull-down assays showed that the circFAM192A targeting probe captured more YTHDF2 than the negative control probe, whereas there was no significant difference in the amount of YTHDC2 captured between the specific and negative control probe (Fig. 4l). RIP assays also confirmed the interaction between circFAM192A and YTHDF2 (Fig. 4j). Moreover, the interaction between circFAM192A and YTHDF2 was strengthened upon FTO knockout but attenuated upon FTO overexpression (Fig. 4k). As reported, HRSP12 may function as a bridge between YTHDF2 and RNase P/MRP to elicit rapid degradation of YTHDF2-bound RNAs [28]. However, silencing POP1 (the key molecule of RNase P) had no effect on circFAM192A expression, implying that YTHDF2 induced circFAM192A degradation was independent of RNase P/MRP (Fig. 4m).

In summary, FTO inhibited circFAM192A decay by eliminating the m^6^A modification in circFAM192A and reducing YTHDF2-dependent recognition.

### CircFAM192A promoted GC proliferation in vitro and in vivo

**Fig. 5:**
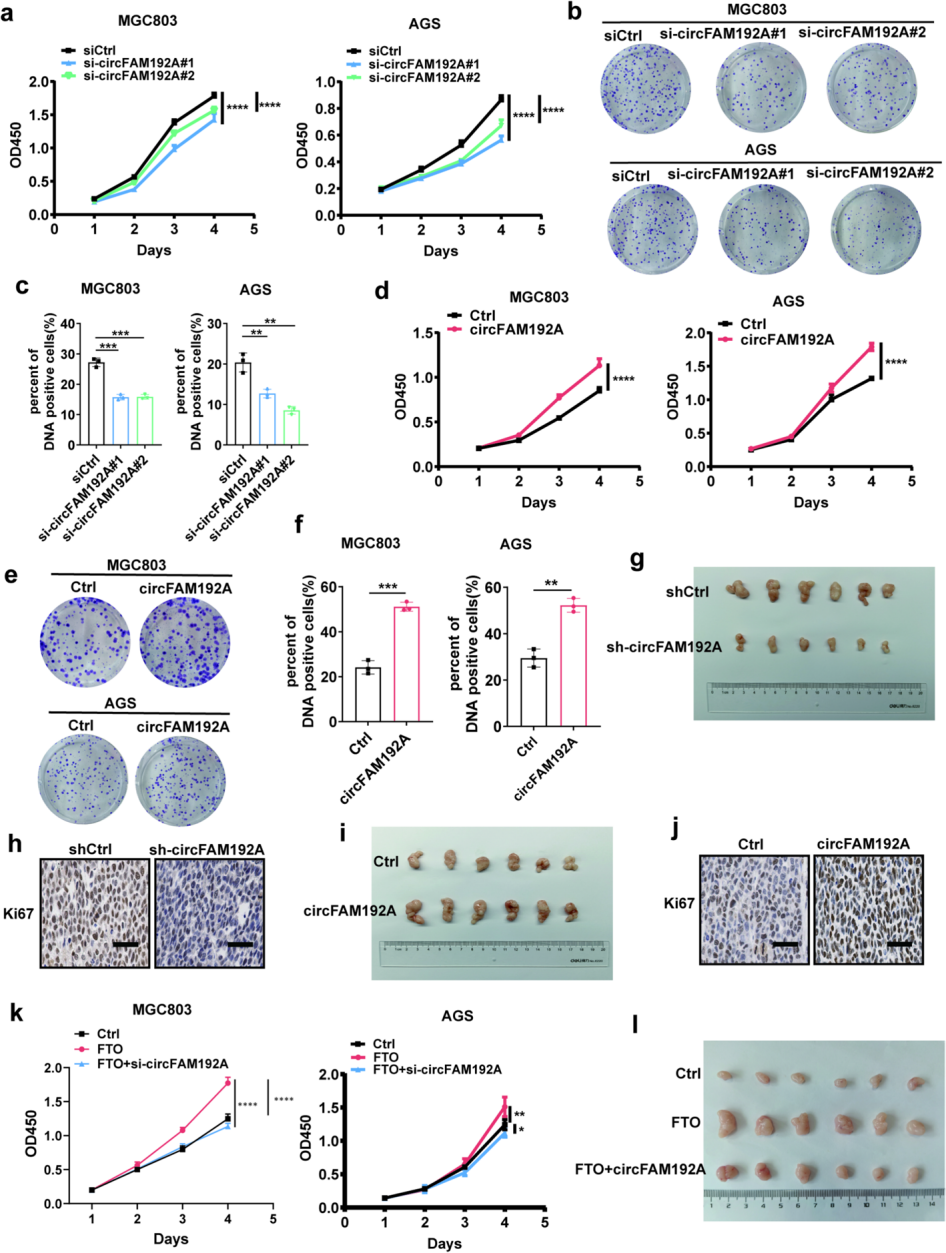
CircFAM192A promoted GC proliferation in vitro and in vivo. **a**-**c** The growth ability of circFAM192A knockdown cells detected by CCK8 (**a**), colony formation (**b**) and EdU experiments (**c**); **d**-**f** The growth ability of circFAM192A overexpressed cells assessed by CCK8 (**d**), colony formation (**e**) and EdU experiments (**f**); **g** Tumors derived from mice injected with circFAM192A knockdown MGC803 cells and controlled cells; **h** IHC assays displayed the Ki67 expression in tumors of the circFAM192A knockdown group, the scale bar is 20 μm; **i** Tumors from mice injected with circFAM192A overexpressed MGC803 cells and controlled cells; **j** IHC staining showed the Ki67 expression in tumors from the circFAM192A overexpressed group, the scale bar is 20 μm; **k** CCK8 assays manifested the growth rate of controlled cells, FTO overexpressed cells, FTO overexpressed but circFAM192A knockdown cells; **l** Tumors from mice injected with controlled cells, FTO overexpressed cells, FTO overexpressed but circFAM192A knockdown cells. Quantitative data from three independent experiments are shown as the mean ± SD (error bars). *P < 0.05, **P < 0.01, ***P < 0.001 (Student’s t-test)

To determine the role of circFAM192A in GC proliferation, we first knocked down circFAM192A expression in GC cells (Fig. S6a). CCK8 assays showed that cells with circFAM192A knocked down exhibited a slower growth rate (Fig. 5a). Cell clones in the circFAM192A knockdown group were fewer and smaller than those in the controlled group (Fig. 5b). Besides, fewer DNA-positive cells were detected when circFAM192A was knocked down (Fig. 5c, Fig. S6c). Opposite results were observed in these experiments when circFAM192A was overexpressed (Fig. 5d-f, Fig. S6b,d).

To confirm the effect of circFAM192A on cell growth in vivo, we injected MGC803 cells with circFAM192A overexpressed or silenced into the left back of nude mice subcutaneously. Results showed that tumors were smaller and lighter with slower growth rate when circFAM192A was knocked down (Fig. 5g, Fig. S6e,f), while tumors with circFAM192A overexpressed grew faster with larger volume and heavier weight (Fig. 5i, Fig. S6g,h). IHC assays also displayed that tumors in circFAM192A knockdown group had a lower level of Ki67 expression and Ki67 in circFAM192A overexpressed group appeared to be higher (Fig. 5h,j). Overall, these findings illustrated that circFAM192A accelerated GC cell proliferation in vitro and in vivo.

Furthermore, to investigate whether FTO promoted GC proliferation through circFAM192A, rescue assays were performed. After knocking down circFAM192A in FTO overexpressed cells, we observed that the ability of FTO to promote cell growth both in vivo and in vitro was weakened after circFAM192A knocked down (Fig. 5k,l, Fig. S7).

### CircFAM192A enhanced SLC7A5 stability and activated the mTOR pathway

**Fig. 6:**
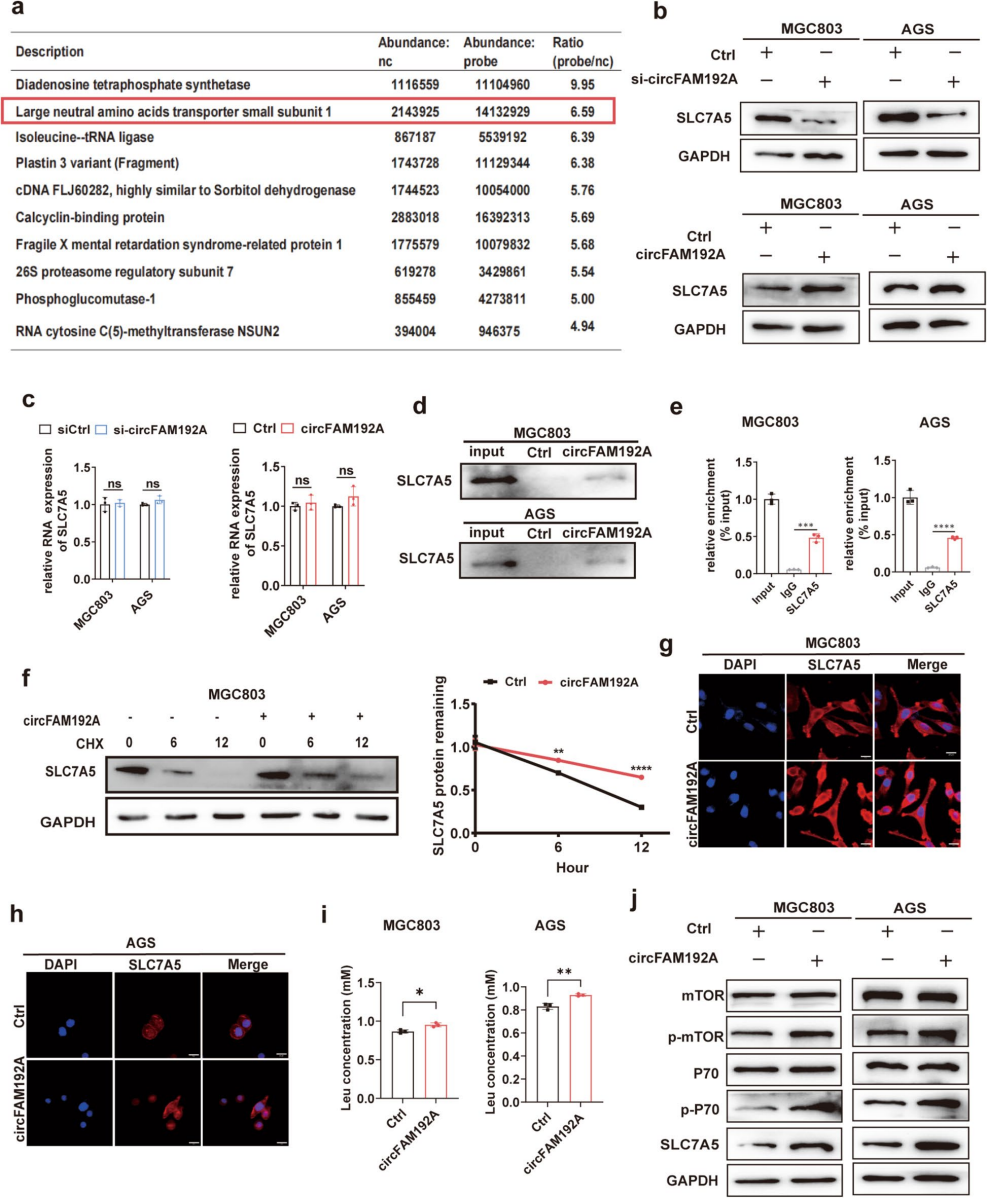
CircFAM192A enhanced the stability of SLC7A5. **a** The top 10 most differentially expressed proteins captured by circFAM192A targeting probes and negative controls; **b** WB assays showed the SLC7A5 expression when circFAM192A was downregulated or upregulated; **c** The level of SLC7A5 mRNA upon circFAM192A was downregulated or upregulated; **d** Pull-down assays revealed the enrichment between SLC7A5 and circFAM192A targeting probes or negative controls; **e** RIP assays showed the level of circFAM192A captured by SLC7A5 or IgG antibody; **f** The remaining SLC7A5 in MGC803 after CHX treatment (100 μg/ml) for 0, 6, 12 h with or without circFAM192A overexpressed; **g**, **h** The images of membrane-located SLC7A5 in cells with circFAM192A upregulated in immunofluorescence assays (scale bar, 20 μm); **i** ELISA assays showed the leucine concentration within cells after circFAM192A upregulated; **j** The alteration of key molecules in mTOR signaling pathway after increasing circFAM192A. Quantitative data from three independent experiments are shown as the mean ± SD (error bars). *P < 0.05, **P < 0.01, ***P < 0.001 (Student’s t-test)

CircRNAs have been reported to function as decoys or sponges for proteins involved in biological processes [29]. We conducted pull-down assays with the circFAM192A targeting probe and the negative probe. The subsequent mass spectrometry displayed differentially expressed proteins between the two group, and the top ten was shown in Fig. 6a. Among these candidates, LAT1 ranked second. LAT1, also named as SLC7A5, was a member of the solute carrier family and in charge of L-leucine transportation. Increased intracellular leucine concentration can activate the mTOR pathway, promoting cellular proliferation and potentially leading to tumorigenesis [19]. Considering its crucial role in cellular activities, SLC7A5 was selected for further investigation.

To figure out whether circFAM192A regulated SLC7A5 expression, we disturbed circFAM192A expression in GC cell lines. Evidently, knocking down circFAM192A led to the downregulation of SLC7A5 and overexpressing circFAM192A caused the elevation of SLC7A5 (Fig. 6b). We then questioned if circFAM192A transcriptionally influenced SLC7A5 expression. However, qPCR assays ruled out the possibility, as disturbing circFAM192A expression had no effect on SLC7A5 mRNA level (Fig. 6c). We then assumed that circFAM192A may exert post-transcriptional regulation on SLC7A5 expression. Pull-down and RIP assays verified the direct interaction between cricFAM192A and SLC7A5 (Fig. 6d,e). We then inhibited the translation process in circFAM192A overexpressed GC cells and untreated cells with CHX. It was evident that circFAM192A had a mitigating effect on SLC7A5 decay (Fig. 6f, Fig. S8a). These results suggested that circFAM192A influenced SLC7A5 expression by impeding its degradation.

SLC7A5 acts as the leucine transporter and increased intracellular leucine concentration activates the mTOR pathway, ultimately promoting cell proliferation. We then tested whether circFAM192A influenced SLC7A5 distribution on the membrane and the leucine concentration within cells. Immunofluorescence assays indicated that the abundance of SLC7A5 on the cell surface was higher in circFAM192A overexpressed cells (Fig. 6g,h). ELISA assays showed that overexpressing circFAM192A caused the leucine accumulation within cells (Fig. 6i). What’s more, through WB assays, we observed that circFAM192A activated the phosphorylation of P70 and mTOR, which are key molecules in the mTOR pathway (Fig. 6j). While knocking down circFAM192A downregulated the location of SLC7A5 on the membrane and inhibited the mTOR pathway (Fig. S8b,c). Above results revealed that SLC7A5 was an oncogenic molecule in GC proliferation and circFAM192A activated the mTOR pathway by inhibited the decay of SLC7A5.

Further, we conducted experiments to ascertain the role of SLC7A5 in GC growth. In vitro assays demonstrated that knocking down SLC7A5 attenuated the proliferative ability of GC cell (Fig. S9a-d). This finding was also observed in in vivo experiments as tumors in the SLC7A5 knockdown group were smaller and lighter with a lower Ki67 expression (Fig. S9e,f).

### FTO promoted GC proliferation through the circFAM192A/SLC7A5 axis

**Fig. 7:**
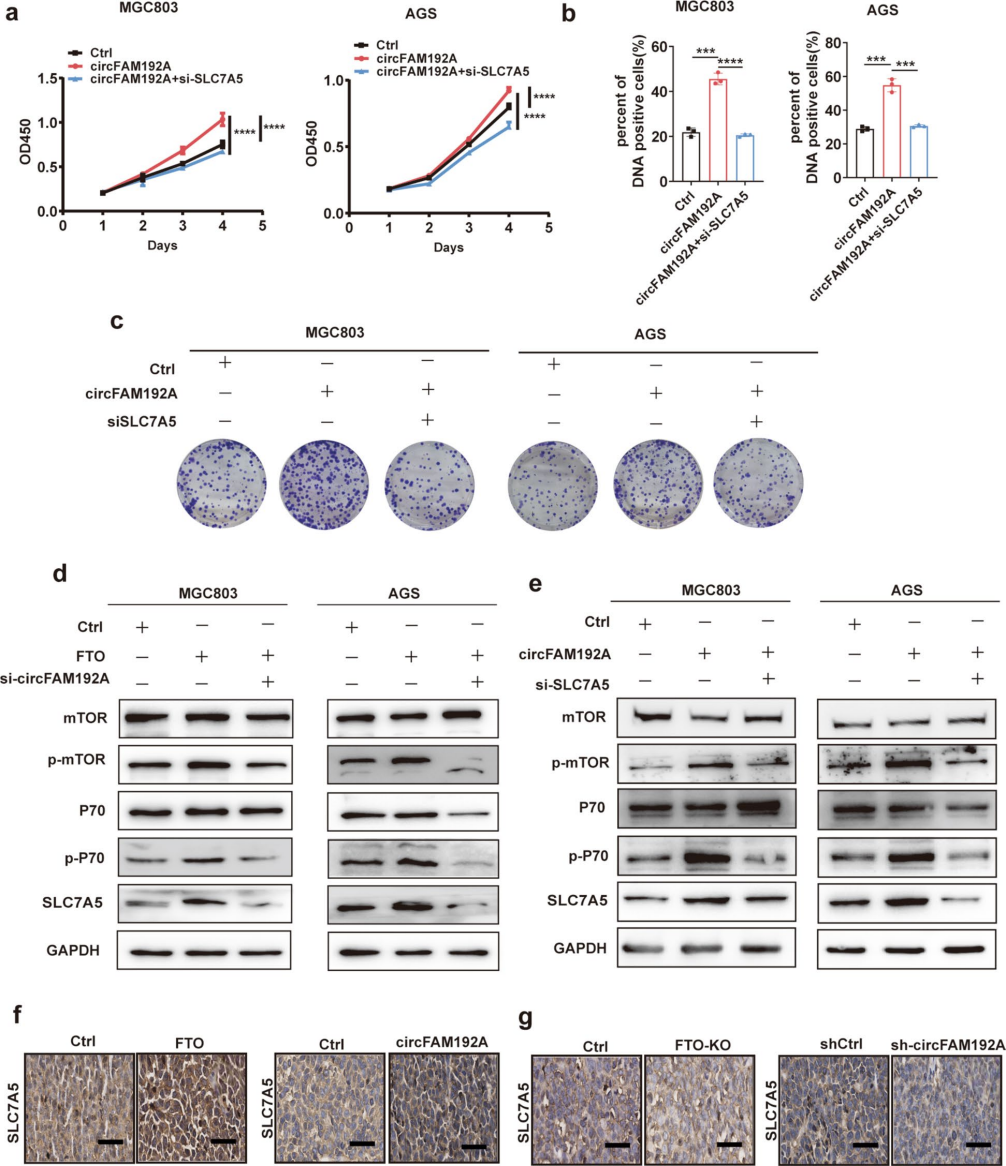
FTO promoted GC proliferation through the circFAM192A/SLC7A5 axis. **a**-**c** CCK8 (**a**), EdU assays (**b**) and colony formation (**c**) assays showed the growth ability of controlled, circFAM192A overexpressed, circFAM192A overexpressed but SLC7A5 silenced MGC803 and AGS cells; **d** WB assays revealed the alteration of key molecules in mTOR signaling pathway in controlled, FTO overexpressed, FTO overexpressed but circFAM192A silenced MGC803 and AGS cells; **e** WB assays revealed the alteration of key molecules in mTOR signaling pathway in controlled, circFAM192A overexpressed, circFAM192A overexpressed but SLC7A5 silenced MGC803 and AGS cells; **f** The level of SLC7A5 in tumors with FTO overexpressed and cirFAM192A overexpressed in IHC assays (scale bar, 20 μm); **g** The expression of SLC7A5 in tumors with FTO knockout and cirFAM192A knockdown in IHC assays (scale bar, 20 μm). Quantitative data from three independent experiments are shown as the mean ± SD (error bars). *P < 0.05, **P < 0.01, ***P < 0.001 (Student’s t-test)

To confirm that FTO promoted GC proliferation through the circFAM192A/SLC7A5 axis, rescue assays were performed. We have observed that silencing circFAM192A weakened the ability of FTO to promote cell growth in CCK8, colony formation and EdU assays (Fig. 5k,l, Fig. S7). Moreover, silencing circFAM192A inhibited the SLC7A5 upregulation and P70/mTOR phosphorylation induced by FTO overexpression (Fig. 7d). Subsequently, we knocking down SLC7A5 in circFAM192A overexpressed cells and found that knocking down SLC7A5 suppressed the effect of circFAM192A on promoting GC cells growth (Fig. 7a-c, Fig. S10) and on mTOR pathway activation (Fig. 7e). What’s more, we conducted IHC assays with tumors obtained from in vivo experiments. IHC assays suggested that the SLC7A5 level was upregulated in tumors from the FTO and circFAM192A overexpressed groups (Fig. 7f) but was downregulated in tumors from the FTO knockout and circFAM192A knockdown groups (Fig. 7g). Generally, a series of rescue assays indicated that FTO promoted GC proliferation through the circFAM192A/SLC7A5 axis.

### FB23-2 suppressed GC proliferation in vitro and in vivo

**Fig. 8:**
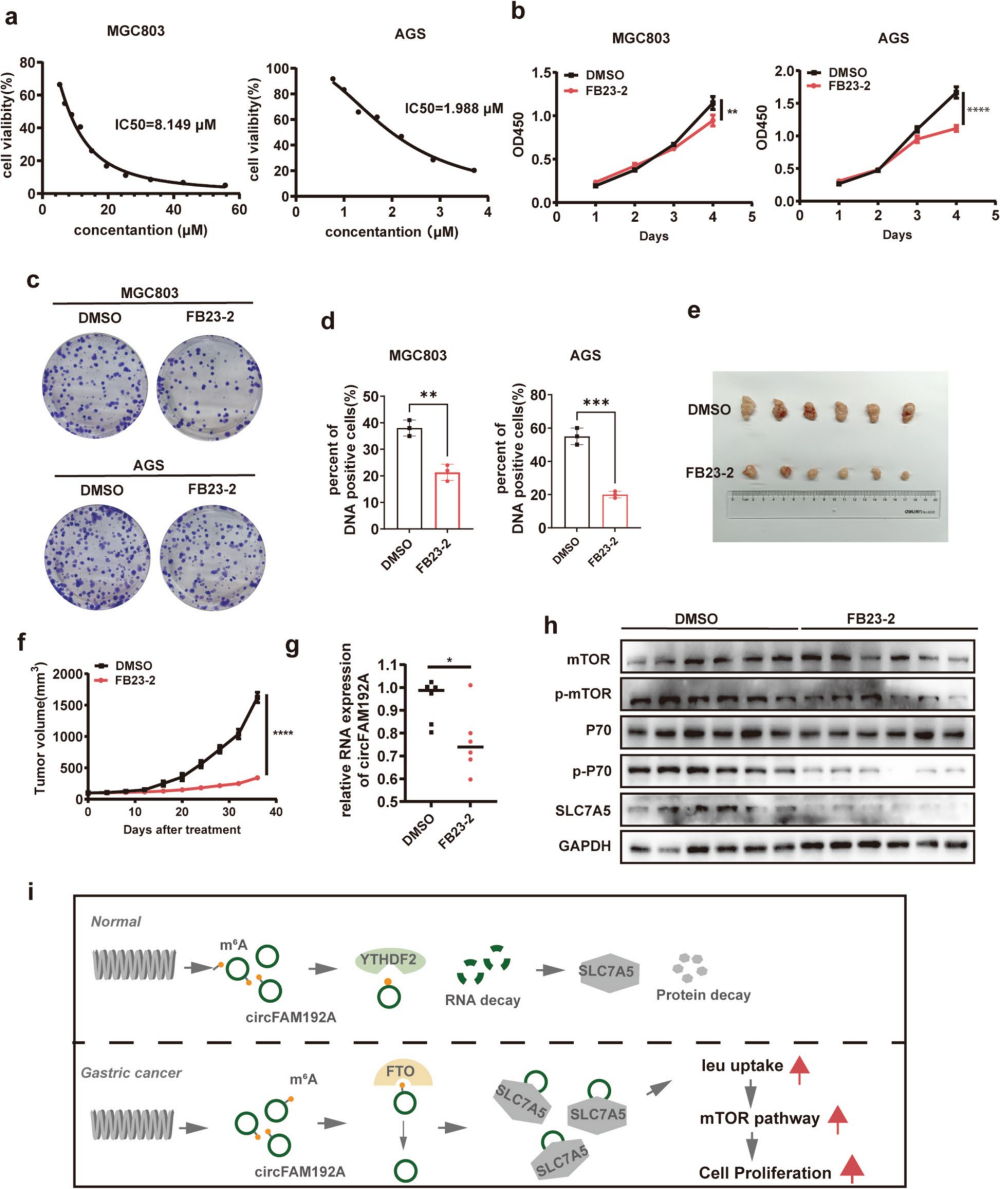
FB23-2 inhibited GC proliferation in vitro and in vivo. **a** The inhibitory effect of FB23-2 with different concentration on MGC803 and AGS cells; (b-d) CCK8 (**b**), colony formation (**c**) and EdU assays (**d**) showed the growth ability of controlled and FB23-2 treated cells (10 μM on MGC803 and 2 μM on AGS); **e** Tumors from mice injected with DMSO and FB23-2 (2 mg/kg); **f** The growth rate of GC cells in vivo after FB23-2 or DMSO treatment; **g** The expression of circFAM192A in FB23-2 or DMSO treated tumors; **h** WB assays showed the expression of SLC7A5, p-p70 and p-mTOR in FB23-2 or DMSO treated tumors; **i** Schematic of the action of FTO regulating gastric cancer proliferation. Quantitative data from three independent experiments are shown as the mean ± SD (error bars). *P < 0.05, **P < 0.01, ***P < 0.001 (Student’s t-test)

FB23-2, the specific inhibitor of FTO, has been reported to inhibit AML progression [30]. After verifying the IC_50_ value of FB23-2 on MGC803 and AGS cells (Fig. 8a), we conducted in vitro experiments with this concentration. Cells with FB23-2 treatment exhibited the weakened growth ability, as shown in cck8, colony formation and EdU assays (Fig. 8b-d, Fig. S11). Furthermore, we generated tumor-bearing models to test FB23-2 efficiency in vivo*.* It was observed that FB23-2 significantly slowed the proliferation of GC (Fig. 8e,f). As FTO promoted GC proliferation through the circFAM192A/SLC7A5 axis, we further conducted qPCR and WB assays with tumors from FB23-2 or DMSO treatment mice model. QPCR assays showed that the expression of circFAM192A was lower in FB23-2 treatment tumors than those in DMSO treatment tumors (Fig. 8g). WB assays showed that SLC7A5 was significantly lower expressed in FB23-2 treatment group and the mTOR pathway was inhibited by FB23-2 (Fig. 8h). Overall, the specific FTO inhibitor, FB23-2, inhibited GC proliferation in vitro and in vivo.

## Discussion

In eukaryotes, gene expression is regulated not only by genomic elements such as promoters and enhancers, but also by chemical modifications in the chromatin and RNAs. Among these modifications, m^6^A is the most prevalent and abundant epigenetic modification in RNAs. This dynamic decoration can be installed by the methyltransferase complex and removed by demethylases. FTO was the first identified demethylase. Numerous studies have demonstrated that FTO played a tumor-promoting role in tumorigenesis, but its role in GC development has received less attention. In our study, we demonstrated that the m^6^A eraser FTO played as an oncogenic role in GC proliferation. First, we identified the increased FTO expression in GC by analyzing data from the TCGA database and our clinical cohort. The increased expression of FTO promoted GC cell proliferation both in vitro and in vivo. Mechanistically, FTO bound to the specific site in circFAM192A and erased the m^6^A modification of circFAM192A. The m^6^A removal increased the stability of circFAM192A and circFMA192A was upregulated in cytoplasm. Then circFMA192A combined with SLC7A5 protein and inhibited its degradation. As a result, more SLC7A5 was located in in the membrane to transport leucine, which activated the mTOR pathway and ultimately accelerated tumor growth (Fig. 8i).

Since then, mounting evidencing suggested that FTO was a tumor-promoting molecule in many cancers, including GC. Our research also showed that FTO promoted GC proliferation, which provided new evidence for the diagnostic role of FTO in tumorigenesis. What’s more, we applied the small-molecule inhibitor of FTO, FB23-2 and found that targeting FTO with FB23-2 could inhibit GC proliferation, which implied that FTO was a promising therapeutic target.

Actually, current studies mainly focused on the relationship between FTO and mRNA. For instance, in acute myeloid leukemia, FTO eradicated the m^6^A modification from ASB2 and RAR-α mRNAs, leading to their degradation and thus suppressing myeloid differentiation [31]. Similarly, FTO-mediated m^6^A removal in MALAT1 mRNAs activated the MALAT1/miR-384/MAL2 cascade, leading to the development of bladder cancer [32]. Whereas, in our study, we identified circFAM192A as the downstream molecule of FTO using MeRIP and RNA sequencing. We further illustrated that FTO erased the m^6^A modification in circFAM192A in the specific site. YTHDF2 was the reader protein that recognized the m^6^A modification in circFAM192A. We demonstrated for the first time how FTO regulated circRNAs in GC development.

SLC7A5 belongs to the SLC super family and is a type of membrane-located transport protein that facilitates the uptake of leucine into cells [33]. Evident studies have implicated SLC7A5 in tumorigenesis through its activation of the mTOR pathway [34]. Our investigation substantiated that SLC7A5 was capable of activating the mTOR pathway in GC cells. Furthermore, we ascertained that circFAM192A directly bound to SLC7A5 and effectively decelerated its rate of degradation.

Though we have demonstrated the role of FTO/circFAM192A/SLC7A5 axis in GC proliferation, there are areas that require further improvement. Firstly, our study was conducted with GC cells and nude mice, it would be more valuable to utilize *Fto*^−/−^ mice with spontaneous GC. On the other hand, FTO may participate in other cellular activities such as immune escape, metabolic reprogramming and so on, which warrant further investigation.

In summary, our study demonstrated that FTO played a pivotal role in promoting GC growth through the circFAM192A/YTHDF2/SLC7A5 axis. Our findings underscored the significant contribution of FTO in GC and its promising therapeutic potential for cancer treatment. Nevertheless, further comprehensive investigations are warranted to fully unravel the enigmatic role of FTO in GC.

## Materials and methods

### Clinical samples and ethics statement

A total of 51 pairs of gastric cancer tissues and adjacent tissues were obtained from the Affiliated People's Hospital of Jiangsu University. Ethical approval in our study was received from Nanjing Medical University (2018-SRFA-074) and Jiangsu University Affiliated People's Hospital (K20180016Y). All studies were conducted under recognized ethical guidelines.

### Cell culture

All cell lines were purchased from the Chinese Academy of Sciences (Shanghai, China). HEK-293 T and MGC803 cells were cultured in RPMI 1640 medium (BI, Iseral) and AGS was cultured in F-12 k medium (Basalmedia, China) with 10% FBS (BI), and 100 ug/ml streptomycin, 100 U/ml penicillin. All the cell lines were incubated under the condition of 37℃ and 5% CO2.

### Quantitative real-time PCR (qRT-PCR) 

qRT-PCR were conducted with the AceQ qPCR SYBR Green Master Mix Kit (Vazyme, China) according to the manufacture’s introductions. All genes were normalized to GAPDH. Primers uesd are shown in Table S1.

### Plasmid/siRNA transfection and lentiviral transduction

The lentivirus vector LV011 overexpressing FTO was generated by Hanbio (Shanghai, China). The lentivirus vector GV392 knocking out FTO and the plasmid GV727 overexpressing circular form of circFAM192A were purchased from by Genechem (Shanghai, China). SiRNAs targeting circFAM192A were obtained from RiboBio (Guangzhou, China). SiRNAs targeting SLC7A5 were obtained from Tsingke Biotechnology Co (Beijing, China). The lentivirus vectors were transfected into cells with polybrene and plasmids, siRNAs were transfected into cells with Lipofectamine 3000 (Thermo). Stable cell lines were selected with puromycin (1ug/ml). SiRNA sequences are followed: si-circFAM192A, GACAAGAATCAAGATTGGT (5’-3’), CAAGAATCAAGATTGGTTG (5’-3’); si-SLC7A5, GGGUGAUGUGUCCAAUCUA (5’-3’), GUGUGAUGACGCUGCUCUA (5’-3’).

### RNA stability assay

Cells were treated with 1 μg/ml actinomycin D and total RNAs were collected at 0, 6, 12 h. RNA level was detected using qRT-PCR, and the halflife of cirRNAs and mRNAs was evaluated.

### RNA fluorescence in situ hybridization (FISH) 

FISH assays were performed using RiboTM Fluorescence In Situ Hybridization Kit (RiboBio) under the manufacturer’s instructions. Cy3-labeled probes targeting circFAM192A, U6, 18S were purchased from RiboBio.

### Ethynyl-2′-deoxyuridine (EdU) incorporation assay

EdU assays were conducted with the Cell-Light EdU DNA Cell Proliferation Kit (RiboBio) according to the manufacturer’s instruction.

### Western blotting (WB) 

GAPDH was used as the internal control. Primary Antibodies included mouse anti-FTO (1:1000, Abcam, USA), mouse anti-SLC7A5 (1:50, santa curz, USA), rabbit anti-YTHDC2 (1:1000, proteintech, China), mouse anti-YTHDF2 (1:1000, proteintech), rabbit anti-mTOR (1:1000, CST), rabbit anti-p-mTOR mouse (1:1000, CST), rabbit anti-p-p70 (1:1000, CST), antiGAPDH (1:20000, Beyotime). Secondary antibodies (A0208 and A0216, Beyotime) were diluted in 1:1000.

### Northern blotting (NB) 

DNA was separated using 1% agarose gel electrophoresis for 20 min under 110v and was detected by BIO-RAD (BIO-RAD Gel Doc XR + , USA).

### Dual luciferase reporter assay

The wild-type sequence of circFAM192A and mutant sequences which contain predicted binding m^6^A site were subcloned into the luciferase reporter vector GV272 (Genechem). HEK-293 T cells were transfected with luciferase reporter vectors containing wild-type sequence or mutant sequence with or without FTO overexpressed. After 24 h incubation, cells were collected and lysed for luciferase activity measure with a specific microplate reader (Synergy H1, USA). Dual-Luciferase®Reporter (DLR™) Assay System was used according to the manufacturer’s instructions.

### Measurement of Leu concentration

The Leu concentration was measured with the ELISIA kit (YJ579653) purchased from Mlbio (Shanghai).

### RNA–protein immunoprecipitation (RIP) 

The MagnaRIP RNA-Binding Protein Immunoprecipitation Kit (Merk) was employed according to the manufacturer’s instructions.

### RNA pull-down

Biotin-labeled probes targeting the junction site of hsa_circ_0006886 were synthesized by RiboBio. RNA pull-down assays were performed using Pierce™ Magnetic RNA–Protein Pull-Down Kit (Thermo) under the instruction. Probes were mixed with streptavidin magnetic beads, which were then incubated with cell lysates collected with IP lysis buffer (Termo). RNA-Binding proteins were acquired for mass spectrum analysis and western blotting analysis. The sequence of probes for circFAM192A were shown: ACAACCAATCTTGATTCTTG-/3bio/;GAAACAACCAATCTTGATTC-/3bio/; CCAATCTTGATTCTTGTCAT-/3bio/

### Animal studies

All animal experiments were approved by the Institutional Animal Care and Use Committee of Nanjing Medical University (IACUC-1706007–3). The 4-week-old female nude mice were divided into different groups randomly and each group consisted of 6. Different treated MGC803 cells (5 × 10^6^ /100 μl) were injected subcutaneously into the left back of mice. when the largest tumor grew nearly 2000mm^3^, mice were sacrificed and tumors were dissected and measured. Notably, in FB23-2/DMSO groups, FB23-2/DMSO was injected intraperitoneally everyday for 2 weeks.

### Bioinformatics analysis

The RNA-seq transcriptome data of 414 stomach adenocarcinoma samples and 36 adjacent normal tissues were obtained from the TCGA data portal (http://portal.gdc.cancer.gov/), and 173 donated normal tissues were from the GTEx database (https://gtexportal.org/home/). Raw read counts were converted to TPM values to make FTO mRNA comparable among different samples.

The OS and PFS information of the TCGA-STAD cohort were obtained from the TCGA data portal, and patients lost to follow-up were excluded. All the RNA-seq and microarray data included in this study were normalized and log2 transformed. The R package “survminer” was used to determine the optimal cutoff value and plot the Kaplan–Meier curves.

### Statistical analysis

All experiments were repeated at least three times independently and results were shown as the means ± SD. Experimental statistical analysis was conducted with The GraphPad Prism 7 software (California, USA). Student’s t-test was applied for single comparison between two groups. The criteria (*p* value < 0.05) was used to determine significance of each experiment (*p < 0.05, **p < 0.01, ***p < 0.001, ****p < 0.0001).

### Supplementary Information

**Additional file 1: Fig. S1.** FTO was upregulated in gastric cancer. (a) qPCR assays showed the level of METTL3 and ALKBH5 in 51 pairs of clinical samples; (b) The expression of METTL3 and ALKBH5 in STAD with GEPIA online tool; (c) WB assays showed the FTO level in 51 pairs of clinical samples. Samples marked with * meant that FTO was highly expressed in the tumor tissue than the paired normal tissue.** Fig. S2.** FTO promoted GC cells proliferation. (a) WB assays confirmed the efficiency of FTO konckout in MGC803 and AGS cell lines; (b) WB assays confirmed the establishment of stable FTO overexpressed cell lines; (c) Bar chart showed clone numbers grown from FTO overexpressed and controlled cells; (d) Bar chart showed clone numbers grown from FTO knockout and controlled cells; (e) The DNA positive cells in FTO overexpressed or controlled cell lines in EdU experiments; (f) EdU assays exihibited the DNA positive cells in FTO knockout and controlled cell lines; (g) The weight and volume of tumors when mice were sacrificed in FTO knockout and controlled group; (h) The growth rate of FTO knockout MGC803 cells and controlled cells *in vivo*; (i) The weight and volume of tumors when mice were sacrificed in FTO overexpressed and controlled group; (j) The growth rate of FTO overexpressed MGC803 cells and controlled cells *in vivo. *Quantitative data from three independent experiments are shown as the mean±SD (error bars). *P < 0.05, **P < 0.01, ***P < 0.001 (Student’s t-test).** Fig. S3.** FTO had no effect on cell migration. (a) Transwell assays showed that there was no significant difference of migrated cell numbers between FTO knockout or control groups; (b) Transwell assays showed that there was no significant difference of migrated cell numbers between FTO overexpressed or control groups; (c) Wound healing assays showed that there was no significant difference of wound areas between FTO knockout or negative control groups; (d) Wound healing assays showed that there was no significant difference of wound areas between FTO overexpressed or negative control groups. Quantitative data from three independent experiments are shown as the mean±SD (error bars). *P < 0.05, **P < 0.01, ***P < 0.001 (Student’st-test).** Fig. S4.** Knocking out FTO influenced the global m6A and circRNA expression pattern. MeRIP seq (a) showed the global alteration of m6A and circRNA seq (b) showed the global alteration of cirRNAs.** Fig. S5.** FTO regulated circFAM192A expression through m6A dependent manner. (a) qPCR assays showed the expression of FTO after knocking down or overexpressing circFAM192A; (b) The sequence of circFAM192A and predicted m6A sites (in red); (c) The graphic structure of luciferase plasmid.** Fig. S6.** CircFAM192A promoted GC proliferation *in vitro and in vivo*. (a) The efficiency of silencing circFAM192A in MGC803 and AGS cells validated by qPCR; 8 (b) The efficiency of overexpressing circFAM192A in MGC803 and AGS cells validated by qPCR; (c) EdU images of circFAM192A knockout and controlled cell lines, the scale bar is 100μm; (d) EdU images of circFAM192A overexpressed and controlled cell lines, the scale bar is 100μm; (e) The weight and volume of tumors when mice were sacrificed in circFAM192A knockdown and controlled group; (f) The growth rate of circFAM192A knockdown MGC803 cells and controlled cells *in vivo*; (g) The weight and volume of tumors when mice were sacrificed in circFAM192A overexpressed and controlled group; (h) The growth rate of circFAM192A overexpressed MGC803 cells and controlled cells *in vivo. *Quantitative data from three independent experiments are shown as the mean±SD (error bars). *P < 0.05, **P < 0.01, ***P < 0.001 (Student’s t-test).** Fig. S7.** FTO promoted GC proliferation dependent of circFAM192A. Colony formation (a) and EdU (b) assays showed the proliferation ability of controlled cells, FTO overexpressed cells, FTO overexpressed but circFAM192A knockdown cells.** Fig. S8.** CircFAM192A enhanced SLC7A5 stability. (a) The remaining SLC7A5 in AGS cells after CHX treatment (100μg/ml) for 0, 6, 12 hours with or without circFAM192A overexpressed; (b) The image of membrane-located SLC7A5 in MGC803 and AGS cells with circFAM192A knockdown in immunofluorescence assays (scale bar, 20μm); (c) The alteration of key molecules in mTOR signaling pathway after knocking down circFAM192A. Quantitative data from three independent experiments are shown as the mean±SD (error bars). *P < 0.05, **P < 0.01, ***P < 0.001 (Student’s t-test).** Fig. S9.** SLC7A5 promoted GC proliferation *in vitro *and *in vivo*. (a) WB assays confirmed the efficiency of knocking down SLC7A5; (b-d) CCK8 (b), colony formation (c) and EdU (d) assays showed the growth ability of cells when knocking down SLC7A5; (e) Tumors derived from mice injected with SLC75 knockdown MGC803 cells; (f) IHC staining showing the Ki67 expression level in tumors from the SLC7A5 knockdown group (scale bar, 20μm). Quantitative data from three independent experiments are shown as the mean±SD (error bars). *P < 0.05, **P < 0.01, ***P < 0.001 (Student’s t-test).** Fig. S10.** CircFAM192A promoted GC proliferation by regulating SLC7A5. EdU (a) and clony formation (b) assays showed the growth ability of controlled, circFAM192A overexpressed, circFAM192A overexpressed but SLC7A5 knockdown MGC803 and AGS cells. Quantitative data from three independent experiments are shown as the mean±SD (error bars). *P < 0.05, **P < 0.01, ***P < 0.001 (Student’s t-test).** Fig. S11.** FB23-2 suppressed GC proliferation *in vitro. *EdU (a) and colony formation (b) assays showed the growth ability of GC cells with FB23-2 treatment. Quantitative data from three independent experiments are shown as the mean ±SD (error bars). *P < 0.05, **P < 0.01, ***P < 0.001 (Student’s t-test).** Table S1.** Primes used in this study.

## Data Availability

Data available on request from the authors.

## Abbreviations

GC: Gastric cancer
m^6^A: N6-methyladenosine
METTL3: Methyltransferase 3
FTO: Fat mass and obesity-associated protein
ALKBH5: AlkB Homolog 5
YTHDCs,YTHDFs: YTH N6-methyladenosine RNA binding proteins
IGF2BPs: Insulin-like growth factor 2 mRNA-binding proteins
SLC7A5: Solute Carrier Family 7 Member 5
OS: Overall survival
PFS: Progression-free survival
RFS: Recurrence-free survival
MeRIP: RNA immunoprecipitation
