# Supplementary material for: Fat mass and obesity-associated protein (FTO) mediated m6A modification of circFAM192A promoted gastric cancer proliferation by suppressing SLC7A5 decay
Source: Mol Biomed. 2024 Apr 1;5:11. doi: 10.1186/s43556-024-00172-4 (PMC10982225; doi:10.1186/s43556-024-00172-4)
Supplement: Supplementary file 1 — Additional file 1: Fig. S1. FTO was upregulated in gastric cancer. (a) qPCR assays showed the level of METTL3 and ALKBH5 in 51 pairs of clinical samples; (b) The expression of METTL3 and ALKBH5 in STAD with GEPIA online tool; (c) WB assays showed the FTO level in 51 pairs of clinical samples. Samples marked with * meant that FTO was highly expressed in the tumor tissue than the paired normal tissue. Fig. S2. FTO promoted GC cells proliferation. (a) WB assays confirmed the efficiency of FTO konckout in MGC803 and AGS cell lines; (b) WB assays confirmed the establishment of stable FTO overexpressed cell lines; (c) Bar chart showed clone numbers grown from FTO overexpressed and controlled cells; (d) Bar chart showed clone numbers grown from FTO knockout and controlled cells; (e) The DNA positive cells in FTO overexpressed or controlled cell lines in EdU experiments; (f) EdU assays exihibited the DNA positive cells in FTO knockout and controlled cell lines; (g) The weight and volume of tumors when mice were sacrificed in FTO knockout and controlled group; (h) The growth rate of FTO knockout MGC803 cells and controlled cells in vivo; (i) The weight and volume of tumors when mice were sacrificed in FTO overexpressed and controlled group; (j) The growth rate of FTO overexpressed MGC803 cells and controlled cells in vivo. Quantitative data from three independent experiments are shown as the mean±SD (error bars). *P < 0.05, **P < 0.01, ***P < 0.001 (Student’s t-test). Fig. S3. FTO had no effect on cell migration. (a) Transwell assays showed that there was no significant difference of migrated cell numbers between FTO knockout or control groups; (b) Transwell assays showed that there was no significant difference of migrated cell numbers between FTO overexpressed or control groups; (c) Wound healing assays showed that there was no significant difference of wound areas between FTO knockout or negative control groups; (d) Wound healing assays showed that there was no significan [file 43556_2024_172_MOESM1_ESM.pdf]

Fat mass and obesity-associated protein (FTO) mediated m^6A modification of circFAM192A promoted gastric cancer proliferation by suppressing SLC7A5 decay

Xi Wu[1, #], Yuan Fang[1, #], Yunru Gu[1, #], Haoyang Shen[1], Yangyue Xu[1], Tingting Xu[1], Run Shi[1], Duo Xu[1], Jingxin Zhang[2], Kai Leng[3], Yongqian Shu[1, *], Pei Ma[1, 4*]

[1]Department of Oncology, the First Affiliated Hospital of Nanjing Medical University, Nanjing 210029, People's Republic of China

[2]Department of General Surgery, Affiliated People's Hospital of Jiangsu University, Zhenjiang Clinic School of Nanjing Medical University, Zhenjiang, People's Republic of China

[3]Department of Medical Informatics, the First Affiliated Hospital of Nanjing Medical University, Nanjing 210029, People's Republic of China.

[4]Jiangsu Key Lab of Cancer Biomarkers, Prevention and Treatment, Nanjing Medical University, Nanjing, China

[#]These authors contributed equally to this work.

*Correspondence should be addressed to Yongqian Shu (yongqian_shu@163.com); Pei Ma (mapei@njmu.edu.cn).

Figure S1

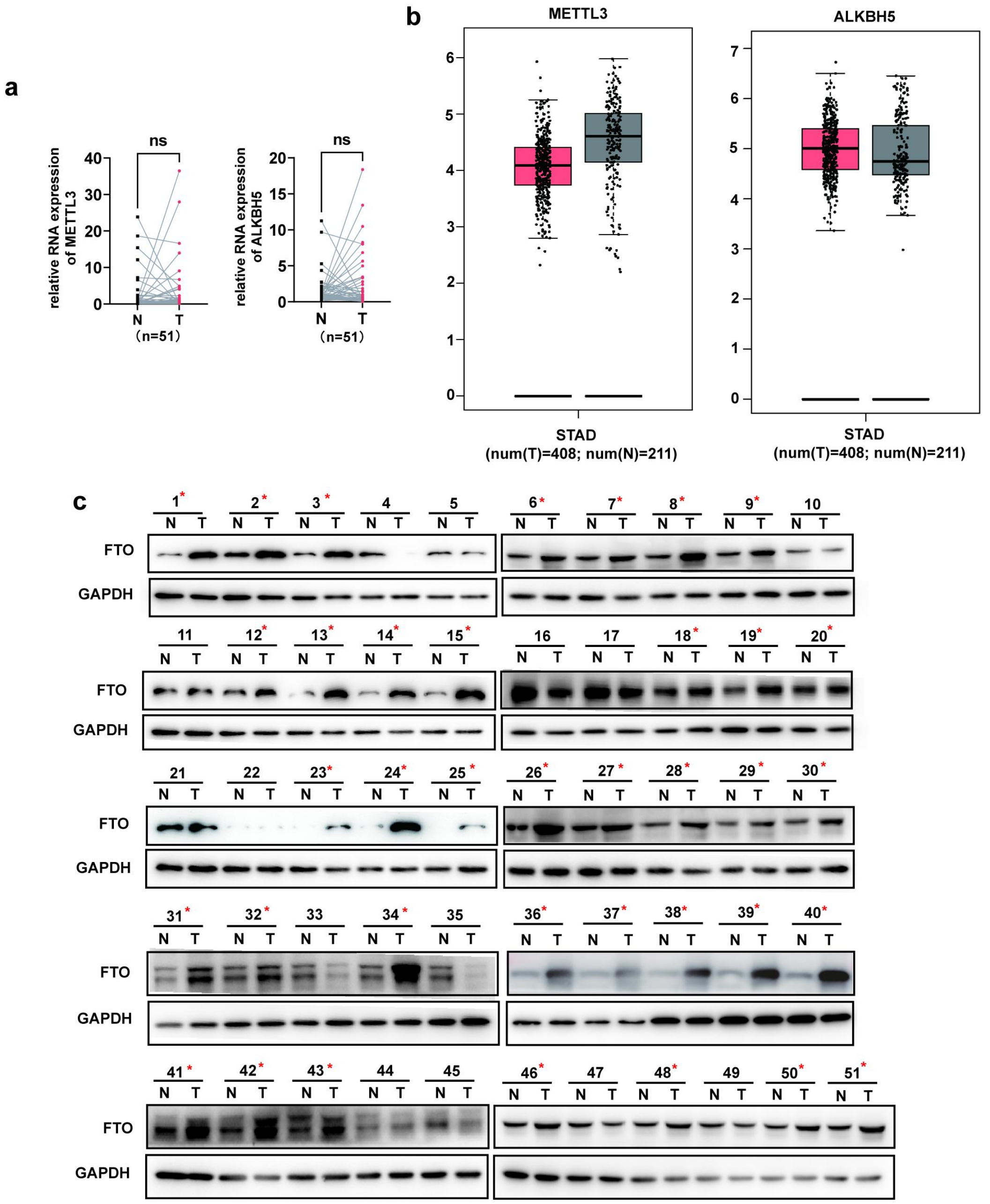

Fig.S1 FTO was upregulated in gastric cancer. (a) qPCR assays showed the level of METTL3 and ALKBH5 in 51 pairs of clinical samples; (b) The expression of METTL3 and ALKBH5 in STAD with GEPIA online tool; (c) WB assays showed the FTO level in 51 pairs of clinical samples. Samples marked with * meant that FTO was highly expressed in the tumor tissue than the paired normal tissue.

Figure S2

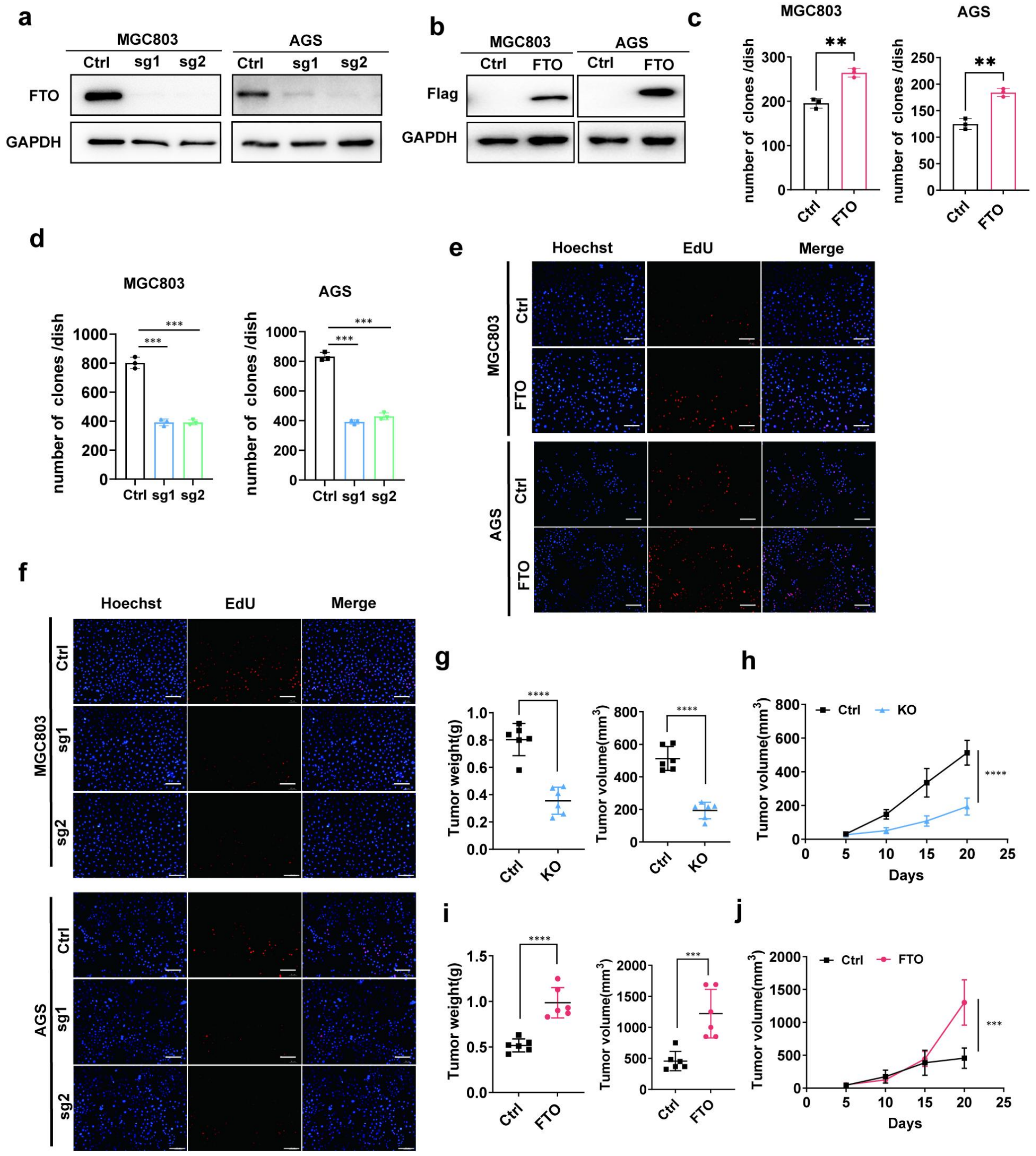

Fig.S2 FTO promoted GC cells proliferation. (a) WB assays confirmed the efficiency of FTO konckout in MGC803 and AGS cell lines; (b) WB assays confirmed the establishment of stable FTO overexpressed cell lines; (c) Bar chart showed clone numbers grown from FTO overexpressed and controlled cells; (d) Bar chart showed clone numbers grown from FTO knockout and controlled cells; (e) The DNA positive cells in FTO overexpressed or controlled cell lines in EdU experiments; (f) EdU assays exihibited the DNA positive cells in FTO knockout and controlled cell lines; (g) The weight and volume of tumors when mice were sacrificed in FTO knockout and controlled group; (h) The growth rate of FTO knockout MGC803 cells and controlled cells *in vivo*; (i) The weight and volume of tumors when mice were sacrificed in FTO overexpressed and controlled group; (j) The growth rate of FTO overexpressed MGC803 cells and controlled cells *in vivo*. Quantitative data from three independent experiments are shown as the mean ± SD (error bars). *P < 0.05, **P < 0.01, ***P < 0.001 (Student's t-test).

Figure S3

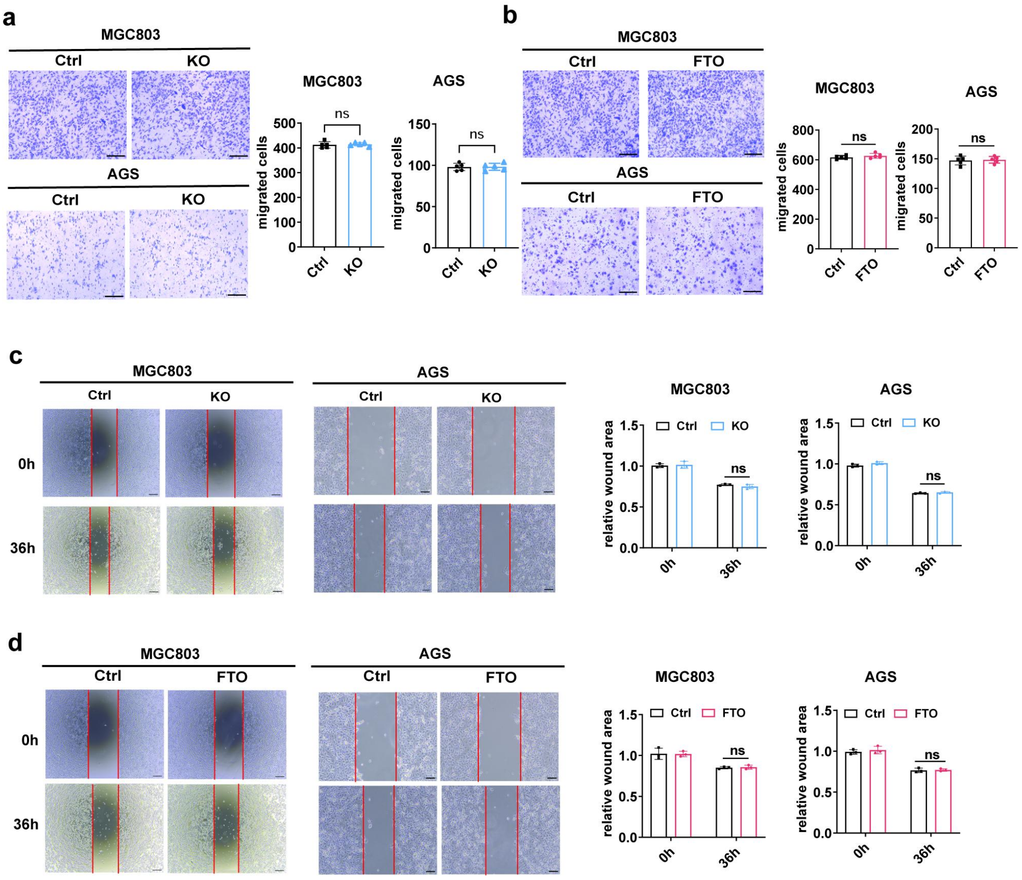

Fig.S3 FTO had no effect on cell migration. (a) Transwell assays showed that there was no significant difference of migrated cell numbers between FTO knockout or control groups; (b) Transwell assays showed that there was no significant difference of migrated cell numbers between FTO overexpressed or control groups; (c) Wound healing assays showed that there was no significant difference of wound areas between FTO knockout or negative control groups; (d) Wound healing assays showed that there was no significant difference of wound areas between FTO overexpressed or negative control groups. Quantitative data from three independent experiments are shown as the mean ± SD (error bars). *$P < 0.05$, **$P < 0.01$, ***$P < 0.001$ (Student's t-test).

Figure S4

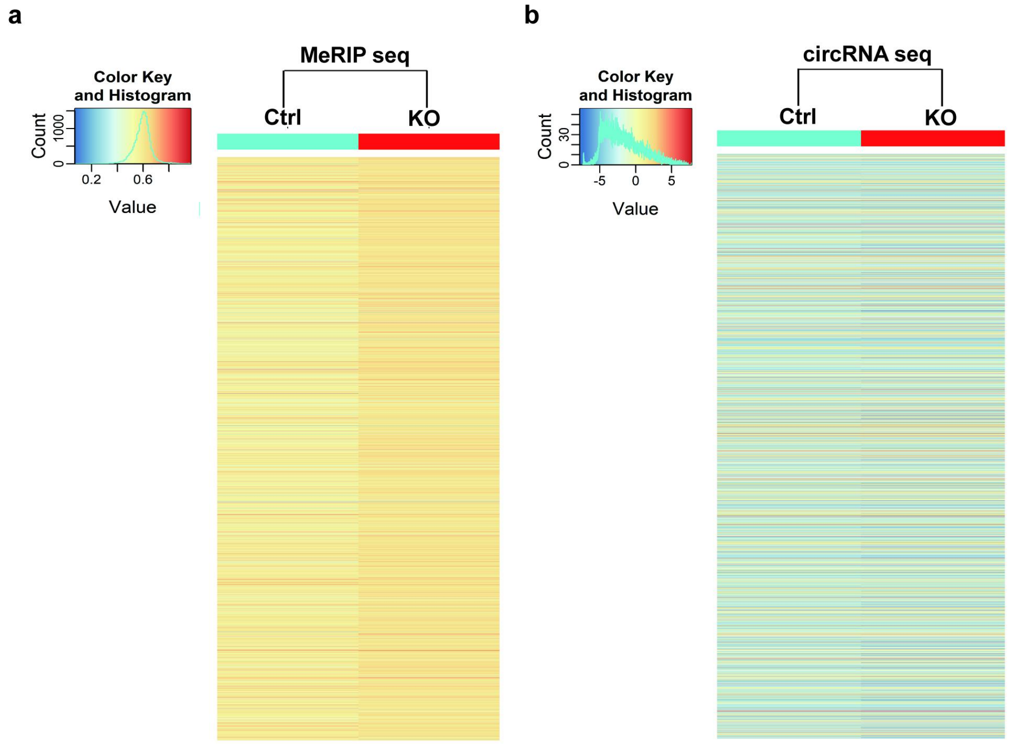

Fig.S4 Knocking out FTO influenced the global m⁶A and circRNA expression pattern. MeRIP seq (a) showed the global alteration of m⁶A and circRNA seq (b) showed the global alteration of cirRNAs.

Figure S5

a

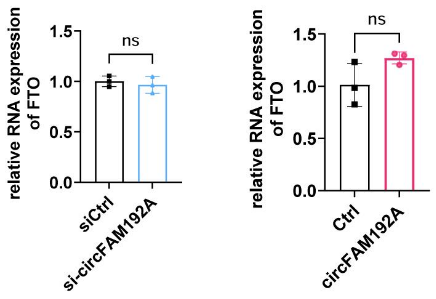

b

>hsa_circ_0006886|NM_024946|FAM192A
ATTGGTTGTTTCATTATGGATGGAGGGGATGATGGTAACCTTATTATCAAAAAGAG-
GTTTGTGTCTGAGGCAGAACTAGATGAACGGCGCAAAAGGAGGCAAGAAGAATGGGAGAAAGTTCGAAAACCT
GAAGATCCAGAAGAATGTCCAGAGGAGGTTTATGACCCTCGATCTCTATATGAAAGGCTACAGGAACAGAAGGA[1]
CAGGAAGCAGCAGGAGTACGAGGAACAGTTCAAATTCAAAAACATGGTAAGAGGCTTAGATGAAGATGAGACCA
ACTTCCTTGATGAGGTTTCTCGACAGCAGGAACTAATAGAAAAGCAACGAAGAGAAGAAGAA[2]CTGAAAGAA[3]CT
GAAGGAATACAGAAATAACCTCAAGAAGGTTGGAATTTCTCAAGAGAACAAGAAGGAAGTGGAAAAGAAACTGA
CTGTGAAGCCTATAGAAACCAAGAACAAGTTCTCCCAGGCGAAGCTGTTGGCAGGAGCTGTGAAGCATAAGAGC
TCAGAGAGTGGCAACAGTGTGAAAAGA[4]CTGAAACCGGACCCTGAGCCAGATGACAAGAATCAAG

c

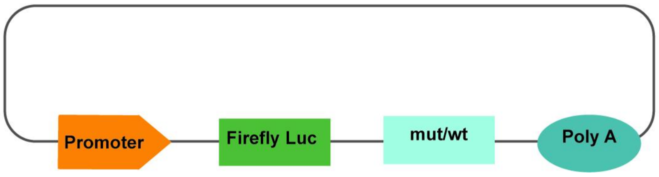

Fig.S5 FTO regulated circFAM192A expression through m^{6}A dependent manner. (a) qPCR assays showed the expression of FTO after knocking down or overexpressing circFAM192A; (b) The sequence of circFAM192A and predicted m^{6}A sites (in red); (c) The graphic structure of luciferase plasmid.

Figure S6

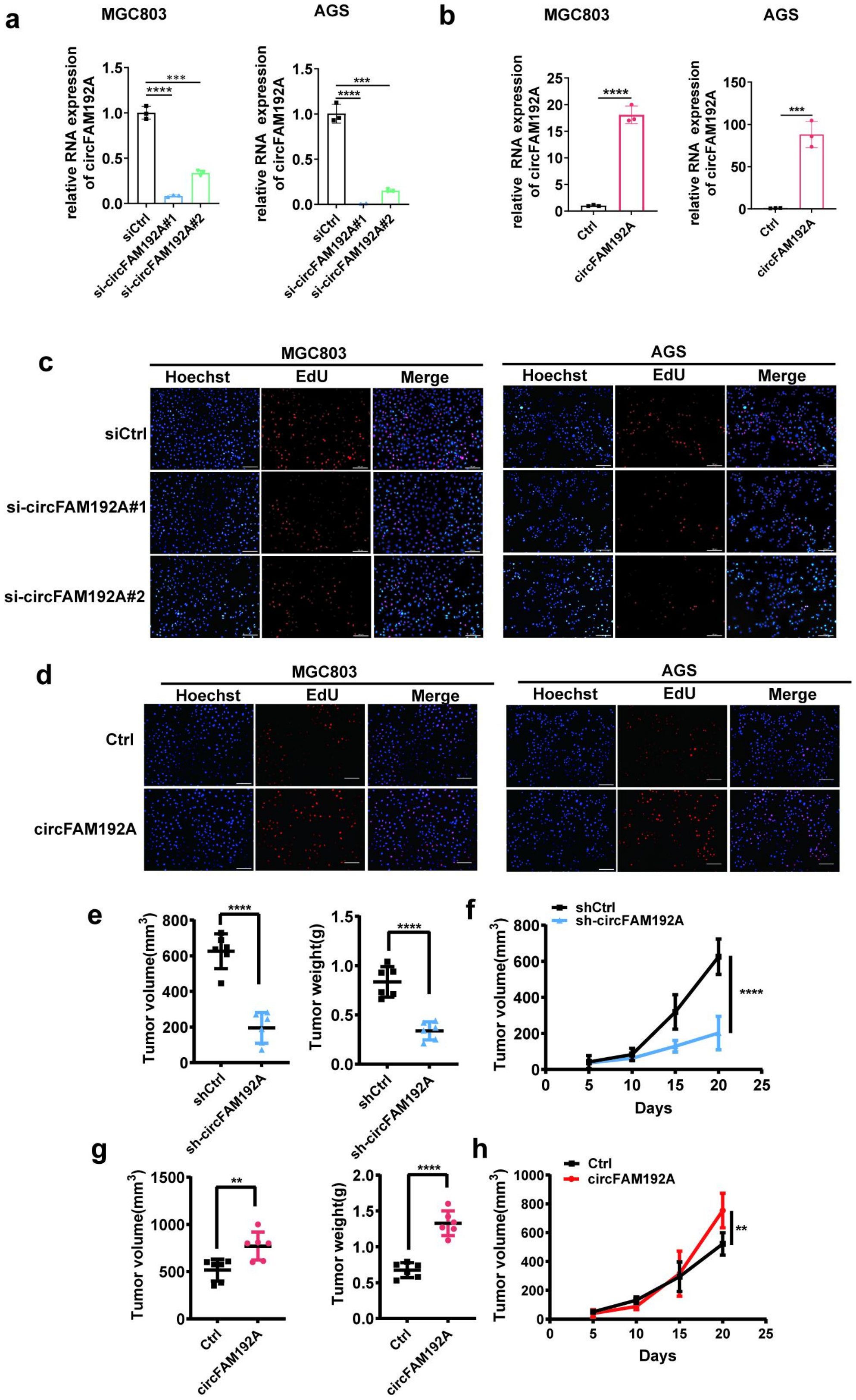

Fig.S6 CircFAM192A promoted GC proliferation *in vitro and in vivo*. (a) The efficiency of silencing circFAM192A in MGC803 and AGS cells validated by qPCR;

(b) The efficiency of overexpressing circFAM192A in MGC803 and AGS cells validated by qPCR; (c) EdU images of circFAM192A knockout and controlled cell lines, the scale bar is 100μm; (d) EdU images of circFAM192A overexpressed and controlled cell lines, the scale bar is 100μm; (e) The weight and volume of tumors when mice were sacrificed in circFAM192A knockdown and controlled group; (f) The growth rate of circFAM192A knockdown MGC803 cells and controlled cells *in vivo*; (g) The weight and volume of tumors when mice were sacrificed in circFAM192A overexpressed and controlled group; (h) The growth rate of circFAM192A overexpressed MGC803 cells and controlled cells *in vivo*. Quantitative data from three independent experiments are shown as the mean ± SD (error bars). *$P < 0.05$, **$P < 0.01$, ***$P < 0.001$ (Student's t-test).

Figure S7

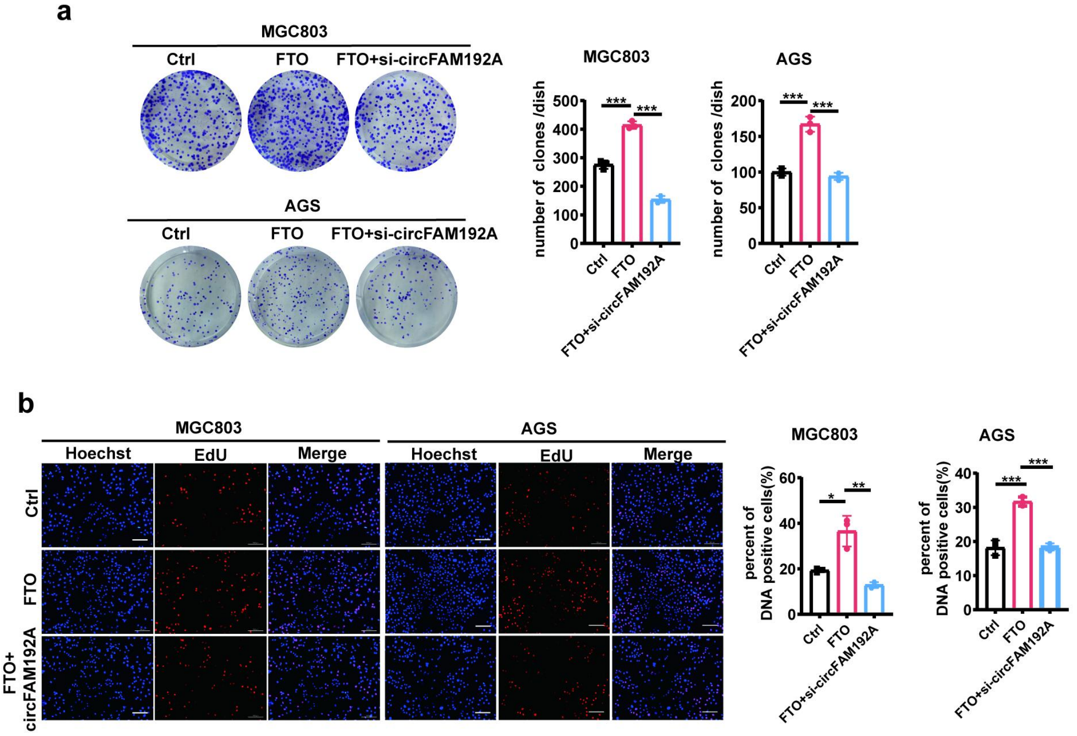

Fig.S7 FTO promoted GC proliferation dependent of circFAM192A. Colony formation (a) and EdU (b) assays showed the proliferation ability of controlled cells, FTO overexpressed cells, FTO overexpressed but circFAM192A knockdown cells

Figure S8

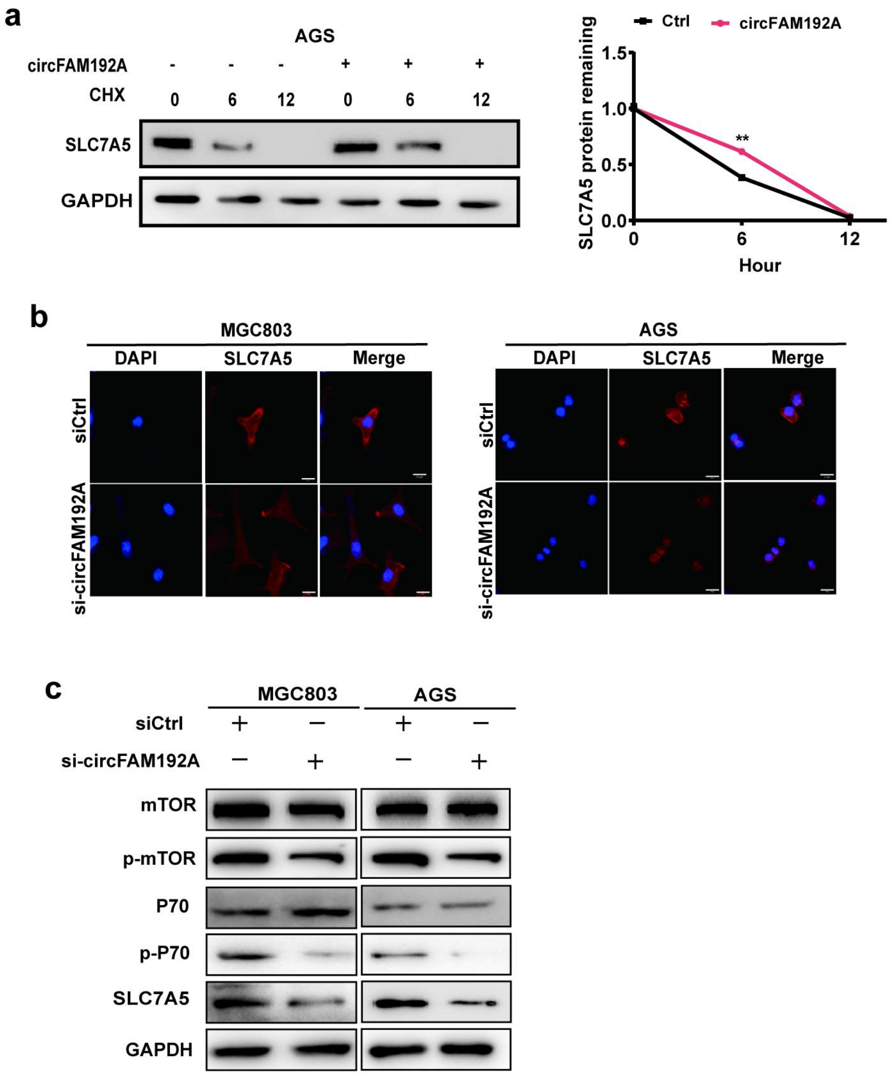

Fig.S8 CircFAM192A enhanced SLC7A5 stability. (a) The remaining SLC7A5 in AGS cells after CHX treatment (100μg/ml) for 0, 6, 12 hours with or without circFAM192A overexpressed; (b) The image of membrane-located SLC7A5 in MGC803 and AGS cells with circFAM192A knockdown in immunofluorescence assays (scale bar, 20μm); (c) The alteration of key molecules in mTOR signaling pathway after knocking down circFAM192A. Quantitative data from three independent experiments are shown as the mean ± SD (error bars). *P < 0.05, **P < 0.01, ***P < 0.001 (Student's t-test).

Figure S9

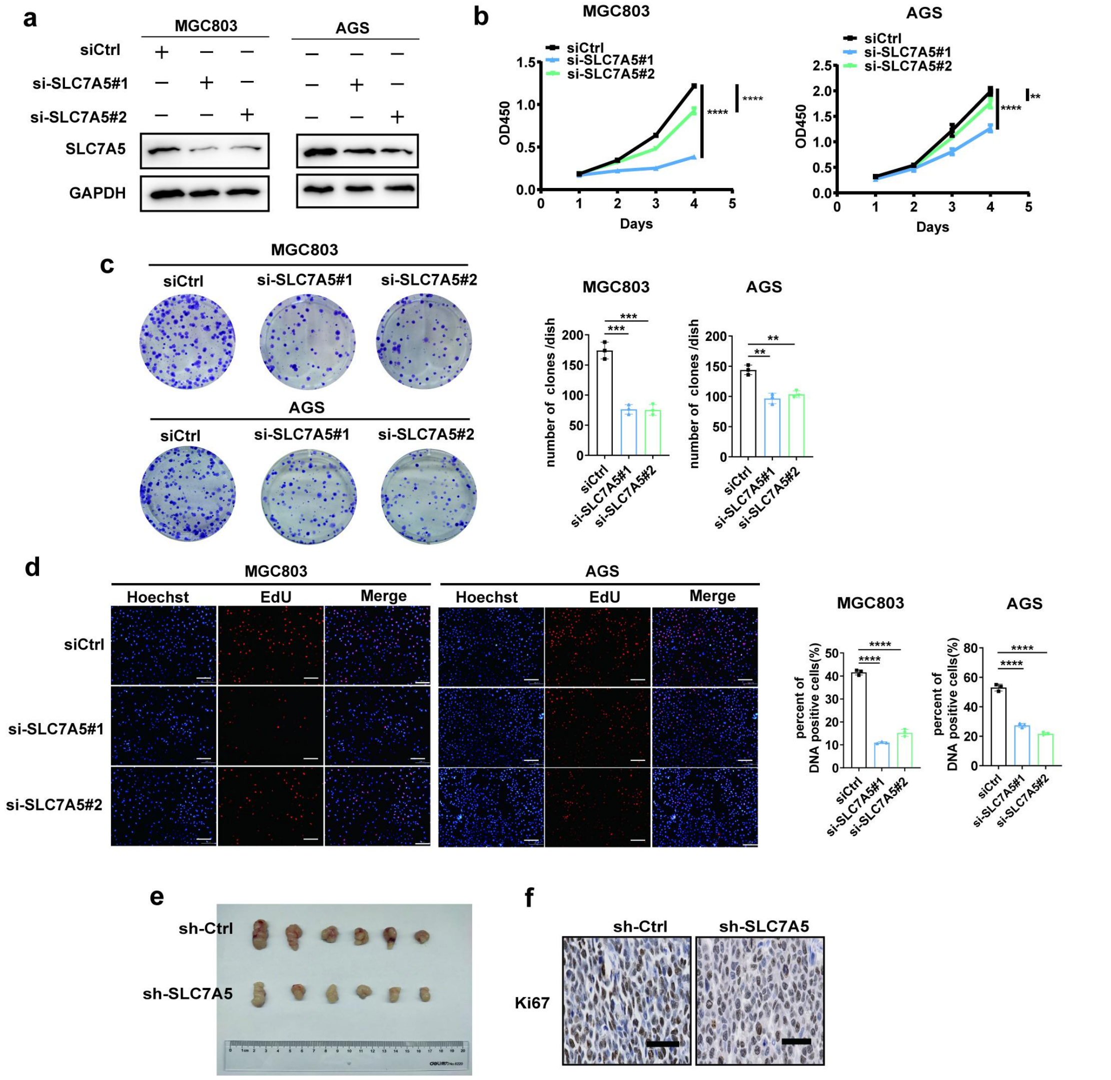

Fig.S9 SLC7A5 promoted GC proliferation *in vitro* and *in vivo*. (a) WB assays confirmed the efficiency of knocking down SLC7A5; (b-d) CCK8 (b), colony formation (c) and EdU (d) assays showed the growth ability of cells when knocking down SLC7A5; (e) Tumors derived from mice injected with SLC75 knockdown MGC803 cells; (f) IHC staining showing the Ki67 expression level in tumors from the SLC7A5 knockdown group (scale bar, 20μm). Quantitative data from three independent experiments are shown as the mean ± SD (error bars). *P < 0.05, **P < 0.01, ***P < 0.001 (Student's t-test).

Figure S10

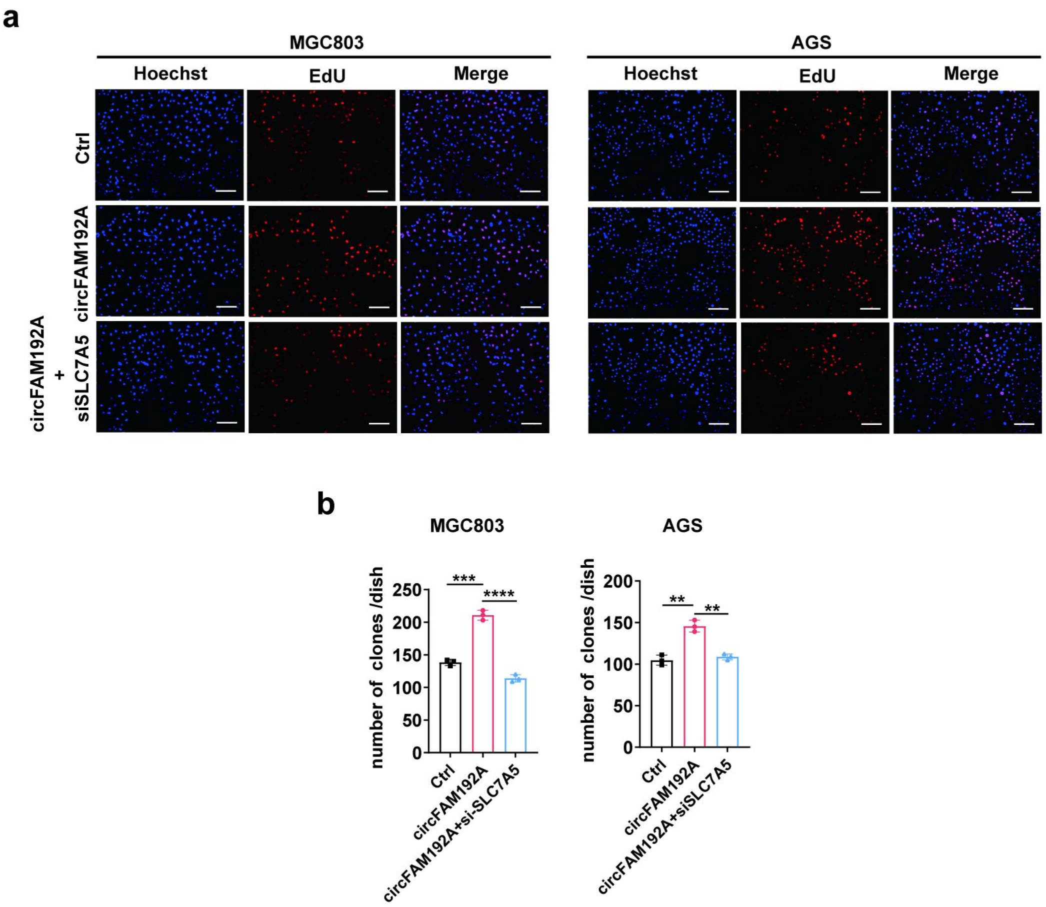

Fig.S10 CircFAM192A promoted GC proliferation by regulating SLC7A5. EdU (a) and clony formation (b) assays showed the growth ability of controlled, circFAM192A overexpressed, circFAM192A overexpressed but SLC7A5 knockdown MGC803 and AGS cells. Quantitative data from three independent experiments are shown as the mean ± SD (error bars). *P < 0.05, **P < 0.01, ***P < 0.001 (Student's t-test).

Figure S11

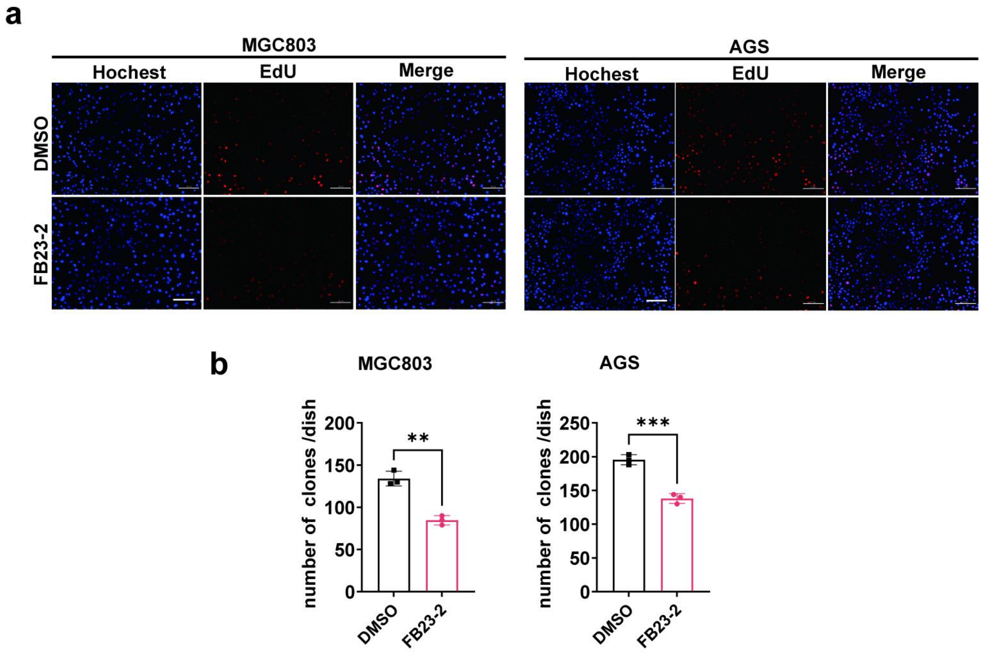

Fig.S11 FB23-2 suppressed GC proliferation *in vitro*. EdU (a) and colony formation (b) assays showed the growth ability of GC cells with FB23-2 treatment. Quantitative data from three independent experiments are shown as the mean ± SD (error bars). *P < 0.05, **P < 0.01, ***P < 0.001 (Student's t-test).

Table S1

Names	Sequences (5'-3')
GAPDH: forward	GGGAGCCAAAAGGGTCAT
GAPDH: reverse	GAGTCCTTCCACGATACCAA
FTO: forward	GAAATAGCCGCTGCTTGTGAGAC
FTO: reverse	CTTGTCCGTTGTAGGATGAACCC
circFAM192A: forward	CCAGATGACAAGAATCAAGATTGGT
circFAM192A: reverse	CCGTTCATCTAGTTCTGCCTCA
FAM192A: forward	GCCAGATGACAAGAATCAAGAGC
FAM192A: reverse	GTTCGGAAGATGGAGGAGACAAT
premRNA of FAM192A: forward	GGGAGGAGTATGGTAAAGTTGGT
premRNA of FAM192A: reverse	GTTGAGGTCCAGAGGAGTTAGATT
divergent primer of circFAM192A: forward	GTTGGCAGGAGCTGTGAAGCATA
divergent primer of circFAM192A: reverse	CTCAGGGTCCGGTTTCAGTCTTT
SLC7A5:forward	CCTCTTTGCCTATGGAGGATGGA
SLC7A5:reverse	ACATGACGCCCAGGTGATAGTTC
POP1:forward	CGCTGGTGGATAGAAACCTGTAA
POP1:reverse	GATGTTATTCCTCCCAACAACTCG

Table S1. Primes used in this study
